# Major Cardiovascular Events After Spontaneous Intracerebral Hemorrhage by Hematoma Location

**DOI:** 10.1001/jamanetworkopen.2023.5882

**Published:** 2023-04-05

**Authors:** Nils Jensen Boe, Stine Munk Hald, Mie Micheelsen Jensen, Line Marie Buch Kristensen, Jonas Asgaard Bojsen, Mohammad Talal Elhakim, Anne Clausen, Sören Möller, Jesper Hallas, Luis Alberto García Rodríguez, Magdy Selim, Larry B. Goldstein, Rustam Al-Shahi Salman, David Gaist

**Affiliations:** 1 Research Unit for Neurology, Odense University Hospital, University of Southern Denmark, Odense, Denmark; 2 Department of Radiology, Odense University Hospital, University of Southern Denmark, Odense, Denmark; 3 Open Patient Data Explorative Network (OPEN), Odense University Hospital, Odense, Denmark; 4 Department of Clinical Research, University of Southern Denmark, Odense, Denmark; 5 Department of Clinical Pharmacology, Pharmacy and Environmental Medicine, University of Southern Denmark, Odense, Denmark; 6 Centro Español Investigación Farmacoepidemiológica, Madrid, Spain; 7 Beth Israel Deaconess Medical Center, Harvard Medical School, Boston, Massachusetts; 8 Department of Neurology and Kentucky Neuroscience Institute, University of Kentucky, Lexington; 9 Centre for Clinical Brain Sciences, University of Edinburgh, Edinburgh, United Kingdom

## Abstract

**Importance:**

Survivors of spontaneous (ie, nontraumatic and with no known structural cause) intracerebral hemorrhage (ICH) have an increased risk of major cardiovascular events (MACEs), including recurrent ICH, ischemic stroke (IS), and myocardial infarction (MI). Only limited data are available from large, unselected population studies assessing the risk of MACEs according to index hematoma location.

**Objective:**

To examine the risk of MACEs (ie, the composite of ICH, IS, spontaneous intracranial extra-axial hemorrhage, MI, systemic embolism, or vascular death) after ICH based on ICH location (lobar vs nonlobar).

**Design, Setting, and Participants:**

This cohort study identified 2819 patients in southern Denmark (population of 1.2 million) 50 years or older hospitalized with first-ever spontaneous ICH from January 1, 2009, to December 31, 2018. Intracerebral hemorrhage was categorized as lobar or nonlobar, and the cohorts were linked to registry data until the end of 2018 to identify the occurrence of MACEs and separately recurrent ICH, IS, and MI. Outcome events were validated using medical records. Associations were adjusted for potential confounders using inverse probability weighting.

**Exposure:**

Location of ICH (lobar vs nonlobar).

**Main Outcomes and Measures:**

The main outcomes were MACEs and separately recurrent ICH, IS, and MI. Crude absolute event rates per 100 person-years and adjusted hazard ratios (aHRs) with 95% CIs were calculated. Data were analyzed from February to September 2022.

**Results:**

Compared with patients with nonlobar ICH (n = 1255; 680 [54.2%] men and 575 [45.8%] women; mean [SD] age, 73.5 [11.4] years), those with lobar ICH (n = 1034; 495 [47.9%] men and 539 [52.1%] women, mean [SD] age, 75.2 [10.7] years) had higher rates of MACEs per 100 person-years (10.84 [95% CI, 9.51-12.37] vs 7.91 [95% CI, 6.93-9.03]; aHR, 1.26; 95% CI, 1.10-1.44) and recurrent ICH (3.74 [95% CI, 3.01-4.66] vs 1.24 [95% CI, 0.89-1.73]; aHR, 2.63; 95% CI, 1.97-3.49) but not IS (1.45 [95% CI, 1.02-2.06] vs 1.77 [95% CI, 1.34-2.34]; aHR, 0.81; 95% CI, 0.60-1.10) or MI (0.42 [95% CI, 0.22-0.81] vs 0.64 [95% CI, 0.40-1.01]; aHR, 0.64; 95% CI, 0.38-1.09).

**Conclusions and Relevance:**

In this cohort study, spontaneous lobar ICH was associated with a higher rate of subsequent MACEs than nonlobar ICH, primarily due to a higher rate of recurrent ICH. This study highlights the importance of secondary ICH prevention strategies in patients with lobar ICH.

## Key Points

**Question:**

Does the risk of major adverse cardiovascular events (MACEs) after an intracerebral hemorrhage (ICH) differ by hematoma location?

**Findings:**

In this cohort study of 2819 patients from 2009 to 2018, compared with patients with nonlobar ICH, those with lobar ICH had significantly higher rates (per 100 person-years) of MACEs (10.84 vs 7.91) and recurrent ICH (3.74 vs 1.24) but not ischemic stroke (1.45 vs 1.77) or myocardial infarction (0.42 vs 0.64).

**Meaning:**

In ICH survivors, lobar hematoma location was associated with an increased risk of MACEs, mostly driven by higher risk of recurrent ICH.

## Teaser

This cohort study of Danish residents examines the risk of major adverse cardiovascular events by hematoma location from an unselected, large cohort of patients with spontaneous intracerebral hemorrhage.

## Introduction

Intracerebral hemorrhage (ICH) is associated with a high short-term case fatality rate,^1^ and survivors have a higher risk of recurrent stroke than population controls.^2,3,4,5^ The location of an ICH can reflect its underlying pathophysiology, with a nonlobar location associated with hypertensive arteriolosclerosis compared with cerebral amyloid angiopathy, which almost exclusively involves lobar locations.^6^

Several studies have investigated the association between hematoma location and the risk of recurrent ICH,^3,7,8,9,10,11,12^ but only a few have also reported the overall risk of other major adverse cardiovascular events (MACEs) in addition to separate risks of ischemic stroke (IS) and myocardial infarction (MI).^3,7,8^ Most of these studies were from single centers^7,10,11^ and, with few exceptions,^10,12^ were relatively small and therefore accrued few outcomes.^7,8,11^

The main objective of the current study was to examine the risk of MACEs by hematoma location from an unselected, large cohort of patients with spontaneous ICH. Our secondary objectives were to investigate the association of hematoma location in patients with 2 established cardiovascular risk factors (previous atrial fibrillation [AF] and diabetes) and previous occlusive vascular disease (ie, IS, MI, or peripheral artery disease)^3^ and to investigate the association between hematoma location and the risk of subsequent stroke (recurrent ICH and IS) and MI.

## Methods

### Setting

The Danish health system is tax funded and free of charge to all residents of the country. This cohort study was conducted in the Region of Southern Denmark (RSD; population of 1.2 million), which is representative of Denmark with respect to demographic characteristics, rural-urban distribution, medication use, and morbidity (eMethods in Supplement 1).^13^ In accordance with Danish law regarding register-based research, the study was approved by the RSD and informed consent was waived. Data were pseudonymized. This study followed the Strengthening and Reporting of Observational Studies in Epidemiology (STROBE) reporting guideline.

### Inception Cohort and Identification of Recurrent Strokes

We defined spontaneous ICH as ICH not attributable to trauma, hemorrhagic transformation of an IS, or an alternative explanation (eg, tumor, cerebrovascular venous sinus thrombosis, vascular malformations, cavernous hemangiomas, or aneurysms).^14^ Using multiple sources, we identified a cohort of all patients 50 years or older hospitalized with first-ever spontaneous ICH (ie, the index ICH) in RSD from January 1, 2009, to December 31, 2018^14,15^ (eMethods in Supplement 1). For each cohort member, we traced all subsequent hospital admissions between the date of the index ICH and the end of the study period (December 31, 2018) that could represent an ICH or IS. We investigated the Danish National Patient Registry^16^ (Patient Registry) for such episodes using both specific and broader discharge diagnosis codes (eMethods in Supplement 1). We also retrieved information from the Danish Stroke Registry^17^ (Stroke Registry) on admissions coded as ICH or IS. For all admissions, whether in the Patient Registry or Stroke Registry, we reviewed medical records (including reports of initial and subsequent brain scans) to classify symptomatic spontaneous stroke events after the index ICH as outlined in the eMethods in Supplement 1.

### Location of ICH

We classified the location of spontaneous symptomatic ICHs (index or recurrent) as nonlobar if the patient had a single infratentorial ICH, a single supratentorial deep ICH (primarily located in the basal ganglia, internal or external capsule, or thalamus), or multiple ICHs in solely nonlobar locations (supratentorial deep or infratentorial); all other ICHs were classified as lobar.^18,19^ We classified location based on information in brain scan reports and discharge summaries, as previously validated.^19^

### Inclusion and Exclusion Criteria

We identified 2819 patients and included those with ICH from the inception cohort^14^ who had a first-ever ICH between January 1, 2009 and November 30, 2018 (patients with ICH onset in December 2018 were excluded to allow for at least 1 month of follow-up) and whose index ICH was classified as lobar or nonlobar. We excluded patients with the index ICH classified as isolated intraventricular hemorrhage, large unclassifiable, or unclassifiable due to missing information.

### Follow-up

In the main analysis, follow-up began from the day of the index ICH and ended on the date of the first outcome event (ie, if a patient first had a recurrent ICH and then an IS, follow-up stopped on date of recurrent ICH) or at censoring (date of death not due to an outcome event, emigration, or end of study [December 31, 2018]), whichever came first. We conducted 2 follow-ups for MACEs and recurrent ICH, IS, and MI. In patients experiencing more than 1 outcome event (or the same type of event occurring more than once) during their follow-up period, only the first outcome was included in the main analysis.

### Outcomes

The primary outcomes were as follows: (1) MACEs (ie, the composite of ICH, IS, spontaneous intracranial extra-axial hemorrhage, MI, systemic embolism, or vascular death); (2) spontaneous recurrent ICH; (3) IS; and (4) MI (defined as discharge primary diagnosis *International Statistical Classification of Diseases and Related Health Problems, Tenth Revision *[*ICD-10*] code of I21, I22, or I23). Vascular death was defined as death within 30 days of a hospital discharge for 1 or more of the events listed in eTable 1 in Supplement 1. Recurrent stroke or intracranial extra-axial hemorrhage events were identified and verified as outlined above and in the eMethods in Supplement 1. Myocardial infarction and the other listed nonstroke events were identified through registry data (ie, admission with primary diagnosis codes of event recorded in the Patient Registry^16^ during follow-up). We used nationwide registry data to establish death (in-hospital or out-of-hospital) within 30 days of any of the listed events.^20^ The health events included under the definition of MACEs are consistent with the definition in previous studies.^3,5^

### Statistical Analysis

We provide baseline (ie, at date of index ICH onset) characteristics of patients with lobar vs nonlobar ICH. We derived figures for the cumulative incidence according to index ICH location (lobar vs nonlobar) and other risk factors at baseline (eg, AF and occlusive vascular disease) using Kaplan-Meier analyses. We also derived corresponding graphs that accounted for competing risk events (Aalen-Johansen estimator).

#### Absolute Event Rates

Under the assumption of a Poisson distribution, we calculated the absolute rates (incidence rates [IRs]; ie, number of events divided by person-years at risk) and corresponding 95% CIs for each outcome within strata defined by index ICH location (lobar vs nonlobar). We also calculated annual IRs for each of the first 5 years of follow-up to assess for variation in the annual risk over time.

#### Relative Event Rates

We used Cox proportional hazards regression models to calculate the hazard ratios (HRs) and corresponding 95% CIs of each outcome (MACEs, recurrent ICH, IS, or MI) in patients with an index lobar ICH compared with a nonlobar index ICH (reference). Proportional hazards assumptions of the Cox models were verified by investigating Schoenfeld residuals. We likewise calculated the risk of each outcome in patients with vs those without comorbid AF, previous occlusive vascular disease, or diabetes overall and, if justified based on sample sizes, stratified by index ICH hematoma location. We calculated adjusted HRs (aHRs) for sex and age (grouped into 50-64 years, 65-74 years, 75-84 years, and ≥85 years) and additional potential confounders (eMethods in Supplement 1) using inverse probability weighting (IPW). We calculated standardized difference of means.^21^ We used the same IPW scores in the main analysis of lobar vs nonlobar locations and in subanalyses by location (eg, AF yes/no stratified by hematoma location). We calculated separate IPW scores for analyses conducted without reference to location (eg, AF yes/no, previous occlusive vascular disease yes/no, and so on). We also performed all analyses with death as a competing event to calculate Fine-Gray subdistribution HRs.

#### Supplementary Analyses

In the main analyses, we calculated the risk of recurrent ICH and IS with censoring follow-up after the first event. We, therefore, did not include an ICH occurring after an IS. We quantified the extent of such multiple outcomes in descriptive supplementary analyses. In the main analysis, follow-up began from the date of the index ICH (day 0). In a supplementary analysis, we began follow-up 31 days after the index ICH, as done in a previous study^22^ because of the high short-term case fatality after ICH. We also calculated risks of recurrent ICH, IS, and MI with follow-up limited to days 0 to 30 after the index ICH. For recurrent stroke occurring during follow-up, we compared case fatality rates on days 1, 7, and 30, and the percentage of patients able to walk unaided after 3 months across outcomes using χ^2^ tests. Assessment of ability to walk was based on all information available in medical records, including both acute admissions and admissions to rehabilitation units. We described event rates for subsequent stroke by extending the follow-up beyond the first recurrent event.

#### Sensitivity Analyses

In the main analyses, we adjusted only for covariates measured at baseline. In a sensitivity analysis of the relative risk for main outcomes after follow-up of the lobar vs nonlobar cohorts, we additionally adjusted for time-dependent covariates corresponding to the use of platelet antiaggregants, oral anticoagulants, statins, and antihypertensive drugs (eMethods in Supplement 1).

#### Validation of Study Method Used to Classify Hematoma Location

We classified the location of the index ICH based on retrieved brain scan reports and discharge summaries^19^ (eMethods in Supplement 1). For a subsample of patients (36% of the cohort), we compared these results with those obtained when using the cerebral hemorrhage anatomical rating instrument CHARTS (Cerebral Haemorrhage Anatomical Rating Instrument)^23^ (eMethods in Supplement 1). We found that our method of classifying hematoma location compared favorably with a classification based on reevaluation of the original brain scans using CHARTS^23^ and masked to clinical details (agreement, 80.4%, κ = 0.68) (eTable 2 in Supplement 1).

Two-tailed *P* < .05 was considered statistically significant. All analyses were performed using Stata software, version 17 (StataCorp LLC). Data were analyzed from February 2022 to September 2022.

## Results

We identified 2819 patients with first-ever spontaneous symptomatic ICH in southern Denmark in 2009 to 2018 of whom 2289 were eligible for this study (eFigure 1 in Supplement 1). We included 1034 patients with lobar ICH (495 men [47.9%] and 539 [52.1%] women; mean [SD] age, 75.2 [10.7] years) and 1255 with nonlobar ICH (680 [54.2%] men and 575 [45.8%] women; mean [SD] age, 73.5 [11.4] years) (Table 1). Further imaging evaluation using magnetic resonance imaging, computed tomography angiography, or both had been performed in 587 patients (56.8%) with lobar ICH and 650 patients (51.8%) with nonlobar ICH. The corresponding percentages for those surviving more than 30 days were 522 patients (73.6%) with lobar ICH and 558 patients (64.3%) with nonlobar ICH.

**Table 1.** Baseline Characteristics of Patients With First-Ever ICH Stratified by Hematoma Location^a^

Characteristic	Crude data			Weighted data		
	Lobar ICH (n = 1034)	Nonlobar ICH (n = 1255)	Mean standardized difference	Lobar ICH (n = 1034)	Nonlobar ICH (n = 1255)	Mean standardized difference	
Age at baseline, mean (SD), y	75.2 (10.7)	73.5 (11.4)	0.1466	74.3 (11.1)	74.3 (11.1)	0.0001	
Sex							
Male	495 (47.9)	680 (54.2)	−0.1265	529.1 (51.2)	643.1 (51.2)	−0.0014	
Female	539 (52.1)	575 (45.8)	0.1265	504.9 (48.8)	611.9 (48.8)	0.0014	
Medical history							
Previous ischemic stroke	130 (12.6)	191 (15.2)	−0.0766	146.9 (14.2)	177.2 (14.1)	0.0025	
Myocardial infarction	56 (5.4)	58 (4.6)	0.0364	50.8 (4.9)	61.9 (4.9)	−0.0013	
Peripheral artery disease	58 (5.6)	78 (6.2)	−0.0257	61.8 (6.0)	74.8 (6.0)	0.0006	
Hypertension	701 (67.8)	920 (73.3)	−0.1211	735.2 (71.1)	890.3 (70.9)	0.0036	
Diabetes	131 (12.7)	174 (13.9)	−0.0512	138.3 (13.4)	168.0 (13.4)	−0.0003	
Atrial fibrillation	216 (20.9)	273 (21.8)	−0.0509	225.7 (21.8)	270.3 (21.5)	0.0070	
COPD	283 (27.4)	373 (29.7)	−0.0324	296.2 (28.6)	358.4 (28.6)	0.0018	
Diagnoses indicative of high alcohol use	87 (8.4)	124 (9.9)	−0.0647	95.0 (9.2)	115.8 (9.2)	−0.0014	
Medication before ICH^b^							
Platelet antiaggregant	381 (36.8)	382 (30.4)	0.1360	342.1 (33.1)	415.8 (33.1)	−0.0011	
Anticoagulant	172 (16.6)	247 (19.7)	−0.0791	191.9 (18.6)	230.8 (18.4)	0.0044	
Statin	310 (30.0)	370 (29.5)	0.0109	305.9 (29.6)	371.4 (29.6)	−0.0003	
Antihypertensives	482 (46.6)	591 (47.1)	−0.0095	485.8 (47.0)	588.5 (46.9)	0.0018	

Abbreviations: COPD, chronic obstructive pulmonary disease; ICH, intracerebral hemorrhage.

^a^ Data are presented as number (percentage) of patients unless otherwise indicated.

^b^ According to data from the Danish National Prescription Registry and corresponding to current use defined as dispensed quantity of drug that lasted until date at onset of index ICH (or ended no later than 30 days before this date).

The baseline prevalence was lower in patients with lobar ICH vs nonlobar ICH for hypertension (701 [67.8%] vs 920 [73.3%]; age- and sex-adjusted odds ratio [aOR], 0.71; 95% CI, 0.59-0.86) and prior IS (130 [12.6%] vs 191 [15.2%]; aOR, 0.78; 95% CI, 0.61-1.00). Use of platelet antiaggregants (381 [36.8%] vs 382 [30.4%]; aOR, 1.31; 95% CI, 1.09-1.57) but not oral anticoagulants (172 [16.6%] vs 247 [19.7%]; aOR, 0.77; 95% CI, 0.62-0.96) was more frequent among patients with lobar than nonlobar ICH. Application of IPW resulted in similar standardized differences of means (Table 1).

### Event Rates by Index ICH Location

During a follow-up of 2048 person-years (mean [SD] follow-up, 2.1 [2.5] years), the frequency of MACEs was higher in the lobar than in the nonlobar cohort (IR, 10.84 [95% CI, 9.51-12.37] vs 7.91 [95% CI, 6.93-9.03]; aHR, 1.26; 95% CI, 1.10-1.44), with a total follow-up of 2780 person-years (mean [SD] follow-up, 2.4 [2.7] years) (Table 2 and Figure; eFigure 2 in Supplement 1). A total of 115 patients had a recurrent ICH, corresponding to crude IRs per 100 person-years of 3.74 (95% CI, 3.01-4.66) for the lobar cohort and 1.24 (95% CI, 0.89-1.73) for the nonlobar cohort (aHR, 2.63; 95% CI, 1.97-3.49). The location of the recurrent ICH was similar to the index ICH in most cases. Among the 1034 patients with lobar index ICH, 70 had a recurrent lobar ICH and 10 a nonlobar ICH. In the 1255 patients with an index nonlobar ICH, 27 had a recurrent nonlobar ICH and 8 had a recurrent lobar ICH. The IRs per 100 person-years and aHRs were similar between the cohorts for IS (IR, 1.45 [95% CI, 1.02-2.06] vs 1.77 [95% CI, 1.34-2.34]; aHR, 0.81; 95% CI, 0.60-1.10) and MI (IR, 0.42 [95% CI, 0.22-0.81] vs 0.64 [95% CI, 0.40-1.01]; aHR, 0.64; 95% CI, 0.38-1.09). eTable 3 in Supplement 1 gives unadjusted HRs and subdistribution HRs.

**Table 2.** Absolute and Relative Rates for Main Outcomes Stratified by Index ICH Hematoma Location and Select Comorbidities

Event during follow-up	Index ICH hematoma location^a^		Atrial fibrillation^b^		Previous occlusive vascular disease^b^^,^^c^		Diabetes^b^	
	Lobar (n = 1034)	Nonlobar (n = 1255)	Yes (n = 489)	No (n = 1800)	Yes (n = 512)	No (n = 1777)	Yes (n = 305)	No (n = 1984)
**Recurrent ICH**								
No. of events/person-years	80/2137	35/2819	11/722	104/4235	23/837	92/4119	6/608	109/4348
Absolute event rate per 100 person-years (95% CI)	3.74 (3.01-4.66)	1.24 (0.89-1.73)	1.52 (0.84-2.75)	2.46 (2.03-2.98)	2.75 (1.83-4.13)	2.23 (1.82-2.74)	0.99 (0.44-2.20)	2.51 (2.08-3.02)
Relative rate, aHR (95% CI)^d^	2.63 (1.97-3.49)	1 [Reference]	0.95 (0.81-1.13)	1 [Reference]	1.76 (1.38-2.24)	1 [Reference]	0.95 (0.74-1.21)	1 [Reference]
**Ischemic stroke**								
No. of events/person-years	31/2137	50/2819	19/722	62/4235	21/837	60/4119	9/608	72/4348
Absolute event rate per 100 person-years (95% CI)	1.45 (1.02-2.06)	1.77 (1.34-2.34)	2.63 (1.68-4.13)	1.46 (1.14-1.88)	2.51 (1.63-4.13)	1.46 (1.13- 1.88)	1.48 (0.77-2.84)	1.66 (1.31-2.09)
Relative rate, aHR (95% CI)^d^	0.81 (0.60-1.10)	1 [Reference]	1.77 (1.44-2.17)	1 [Reference]	1.65 (1.23-2.20)	1 [Reference]	0.53 (0.37-0.76)	1 [Reference]
**Myocardial infarction**								
No. of events/person-years	9/2137	18/2819	8/722	19/4235	8/837	19/4119	0/608	27/4348
Absolute event rate per 100 person-years (95% CI)	0.42 (0.22-0.81)	0.64 (0.40-1.01)	1.11 (0.55- 2.22)	0.45 (0.29- 0.70)	0.96 (0.48-1.91)	0.46 (0.29- 0.72)	0	0.62 (0.43-0.91)
Relative rate, aHR (95% CI)^d^	0.64 (0.38-1.09)	1 [Reference]	3.03 (2.31- 3.96)	1 [Reference]	1.08 (0.61-1.92)	1 [Reference]	NE^e^	1 [Reference]
**MACEs^f^**								
No. of events/person-years	222/2048	220/2780	100/686	342/4142	105/811	337/4017	53/585	389/4243
Absolute event rate per 100 person-years (95% CI)	10.84 (9.51-12.37)	7.91 (6.93-9.03)	14.58 (11.98-17.73)	8.26 (7.43-9.18)	12.95 (10.70-15.68)	8.39 (7.54-9.33)	9.06 (6.92-11.86)	9.17 (8.30-10.13)
Relative rate, aHR (95% CI)^d^	1.26 (1.10-1.44)	1 [Reference]	2.73 (2.55-2.92)	1 [Reference]	1.40 (1.23-1.59)	1 [Reference]	0.96 (0.84-1.10)	1 [Reference]

Abbreviations: aHR, adjusted hazard ratio; ICH, intracerebral hemorrhage; MACE, major adverse cardiovascular event; NE, not estimated.

^a^ Location of hematoma on brain scan of first-ever intracerebral hemorrhage.

^b^ Classified based on information at baseline.

^c^ Medical history of ischemic stroke, myocardial infarction, or peripheral arterial disease at baseline.

^d^ Hazard ratio adjusted for sex, age (<75 years [reference], 75-84, or ≥85 years), hypertension, atrial fibrillation, previous ischemic stroke, myocardial infarction, peripheral arterial disease, diabetes, chronic obstructive pulmonary disease (as a marker of smoking), diagnoses indicative of high alcohol use, and use of medications (separate covariates for each of the following drug classes: platelet antiaggregant [low-dose aspirin or clopidogrel], anticoagulants [direct oral anticoagulants or vitamin K antagonist], antihypertensives, and statins).

^e^ Not estimated because of sparse events.

^f^ Major adverse cardiovascular event defined as stroke (ICH or ischemic stroke), myocardial infarction, systemic embolism, or vascular death.

**Figure.**
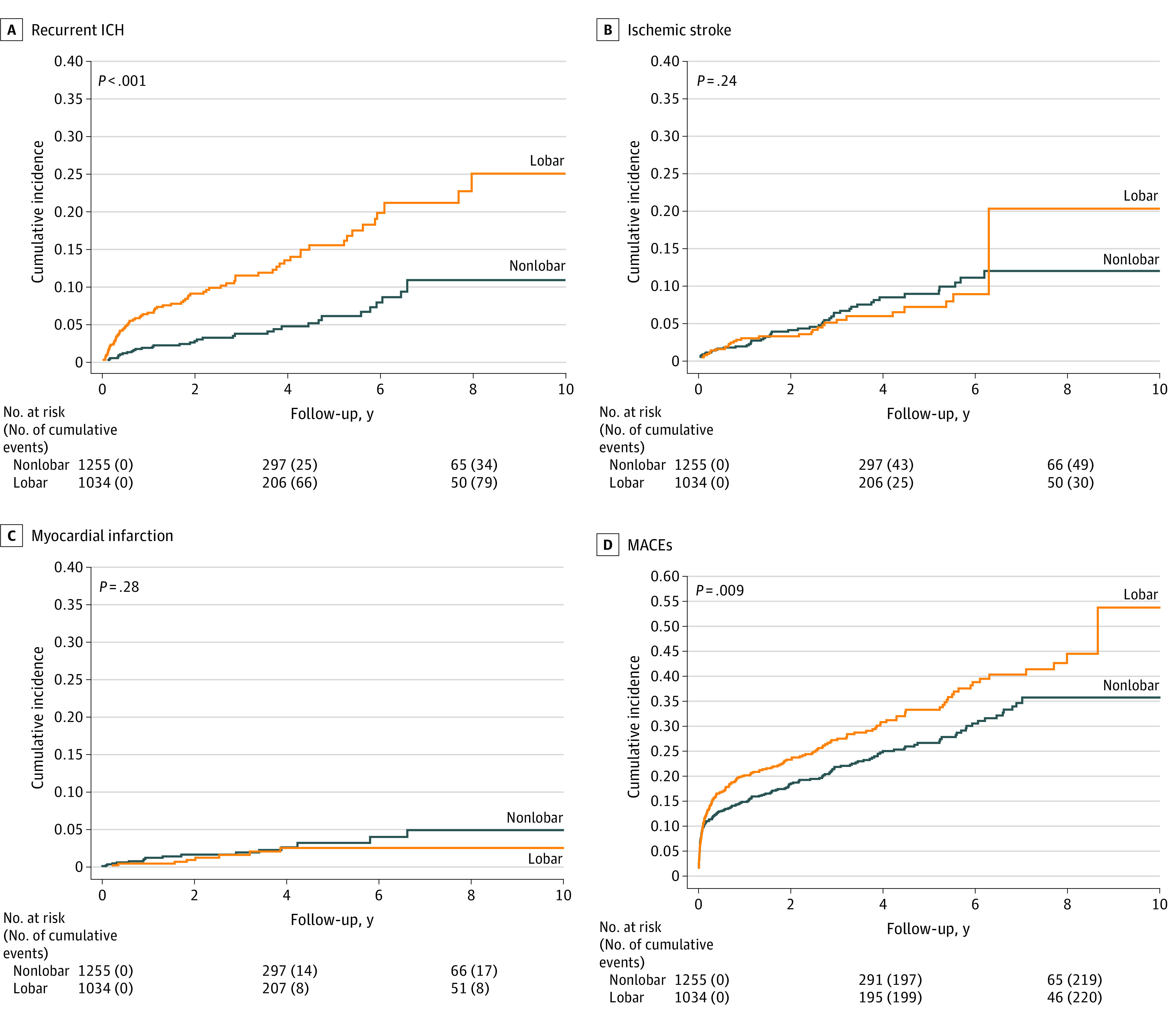
Cumulative Incidence of Main Outcomes by Hematoma Location (Lobar vs Nonlobar) of the Index Intracerebral Hemorrhage (ICH) MACE indicates major adverse cardiovascular event.

### Event Rates by Select Comorbidities

Risks for MACEs, IS, and MI (but not recurrent ICH) were higher among patients with than without AF irrespective of hematoma location (Table 2; eTable 3 in Supplement 1). Risks for all outcomes were higher in patients with than without prior occlusive vascular disease (Table 2; eTable 3 in Supplement 1). Patients with diabetes at ICH onset had similar risks as those without diabetes, except for IS, which occurred less frequently during follow-up in patients with diabetes; because there were few patients with diabetes, we did not pursue further analyses stratified by diabetes status.

### Event Rates Stratified by ICH Location and Select Comorbidities

In patients with baseline (at time of index ICH) comorbid AF, the relative rates of the main outcomes did not differ by hematoma location (Table 3; eTable 4 and eFigure 3 in Supplement 1), although the rate of IS was lower after lobar ICH (aHR, 0.50; 95% CI, 0.26-0.97). In patients without comorbid AF, the risk of recurrent ICH (aHR, 2.91; 95% CI, 2.15-3.95) was higher than the risk of IS (aHR, 0.92; 95% CI, 0.65-1.30) in the lobar cohort, as was the risk of MACEs (aHR, 1.45; 95% CI, 1.25-1.69). Compared with the nonlobar cohort, patients with lobar ICH and no occlusive vascular disease had a higher risk for ICH recurrence (aHR, 2.20; 95% CI, 1.61-3.01). This risk was even higher among patients who had a history of previous occlusive vascular disease (aHR, 6.15; 95% CI, 2.98-12.67) (Table 3; eTable 4 and eFigure 4 in Supplement 1). For subanalyses and sensitivity analyses results, see eResults, eTables 5 to 9, and eFigure 5 in Supplement 1.

**Table 3.** Absolute and Relative Rates of Main Outcomes Stratified by Index ICH Hematoma Location Within Strata of Patients With or Without Atrial Fibrillation or Previous Occlusive Vascular Disease

Event during follow-up	Atrial fibrillation^a^^,^^b^				Previous occlusive vascular disease^a^^,^^b^^,^^c^			
	Yes		No		Yes		No	
	Lobar (n = 216)	Nonlobar (n = 273)	Lobar (n = 218)	Nonlobar (n = 294)	Lobar (n = 218)	Nonlobar (n = 294)	Lobar (n = 816)	Nonlobar (N = 961)
**Recurrent ICH**								
No. of events/person-years	6/345	5/376	74/1792	30/2442	17/267	6/571	63/1871	29/2248
Absolute event rate per 100 person-years (95% CI)	1.74 (0.78-3.87)	1.33 (0.55-3.19)	4.13 (3.29-5.19)	1.23 (0.86-1.76)	6.38 (3.96-10.26)	1.05 (0.47-2.34)	3.37 (2.63-4.31)	1.29 (0.90-1.86)
Relative rate, aHR (95% CI)^d^	1.13 (0.49-2.63)	1 [Reference]	2.91 (2.15-3.95)	1 [Reference]	6.15 (2.98-12.67)	1 [Reference]	2.20 (1.61-3.01)	1 [Reference]
**Ischemic stroke**								
No. of events/person-years	6/345	13/376	25/1792	37/2442	8/267	13/571	23/1871	37/2248
Absolute event rate per 100 person-years (95% CI)	1.74 (0.78-3.87)	3.45 (2.01-5.95)	1.40 (0.94-2.06)	1.51 (1.10-2.09)	3.00 (1.50-6.00)	2.28 (1.32-3.92)	1.23 (0.82-1.85)	1.65 (1.19-2.27)
Relative rate, aHR (95% CI)^d^	0.50 (0.26-0.97)	1 [Reference]	0.92 (0.65-1.30)	1 [Reference]	1.42 (0.77-2.61)	1 [Reference]	0.72 (0.50-1.02)	1 [Reference]
**Myocardial infarction**								
No. of events/person-years^e^	NR	NR	NR	NR	NR	NR	NR	NR
Absolute event rate per 100 person-years (95% CI)	0.87 (0.28-2.70)	1.33 (0.55-3.19)	0.33 (0.15-0.75)	0.53 (0.31 0.92)	0.75 (0.19-3.00)	1.05 (0.47-2.34)	0.37 (0.18-0.78)	0.53 (0.30-0.94)
Relative rate, aHR (95% CI)^d^	NE^f^	1 [Reference]	0.62 (0.31-1.21)	1 [Reference]	0.59 (0.20-1.75)	1 [Reference]	0.72 (0.38-1.39)	1 [Reference]
**MACEs^g^**								
No. of events/person-years	38/319	62/367	184/1728	158/2413	48/241	57/569	174/1806	163/2211
Absolute event rate per 100 person-years (95% CI)	11.91 (8.66-16.36)	16.90 (13.18-21.68)	10.65 (9.21-12.30)	6.55 (5.60-7.65)	19.90 (14.99-26.40)	10.01 (7.72-12.98)	9.63 (8.30-11.18)	7.37 (6.32-8.60)
Relative rate, aHR (95% CI)^d^	0.75 (0.57-0.99)	1 [Reference]	1.45 (1.25-1.69)	1 [Reference]	1.57 (1.20-2.07)	1 [Reference]	1.21 (1.04-1.40)	1 [Reference]

Abbreviations: aHR, adjusted hazard ratio; ICH, intracerebral hemorrhage; MACE, major adverse cardiovascular event; NE, not estimated; NR, not reported.

^a^ Classified based on information at baseline.

^b^ Location of hematoma on brain scan of first-ever intracerebral hemorrhage.

^c^ Medical history of ischemic stroke, myocardial infarction, or peripheral arterial disease at baseline.

^d^ Hazard ratio adjusted for sex, age (<75 [reference], 75-84, or ≥85 years), hypertension, atrial fibrillation, previous ischemic stroke, myocardial infarction, peripheral arterial disease, diabetes, chronic obstructive pulmonary disease (as a marker of smoking), diagnoses indicative of high alcohol use, and use of medications (separate covariates for each of the following drug classes: platelet antiaggregant [low-dose aspirin or clopidogrel], anticoagulants [direct oral anticoagulants or vitamin K antagonist], antihypertensives, and statins).

^e^ Not reported to preserve anonymity in view of small cell counts.

^f^ Not estimated because of sparse events.

^g^ Major adverse cardiovascular event defined as stroke (ICH or ischemic stroke), myocardial infarction, systemic embolism, or vascular death.

## Discussion

In this cohort study of 1034 patients with lobar ICH and 1255 patients with nonlobar ICH, we found that lobar ICH was associated with a higher risk of subsequent MACEs and separately recurrent ICH but not IS or MI. This topographical association was strongest in patients with previous occlusive vascular disease (prior IS, peripheral arterial disease, or MI) and was also found in patients without baseline comorbid AF. In patients with comorbid AF, absolute rates of MACEs and MI but not ICH were higher in the nonlobar than the lobar cohort, with the rate of IS lower in those with lobar ICH, although these differences were not significant.

The location-specific rates of recurrent ICH and IS in this study are similar to those reported in southern England and somewhat lower than those in Lothian, Scotland.^3^ In addition, the adjusted hazard ratio for recurrent ICH by index hematoma location (lobar vs nonlobar) in our study was consistent with the pooled relative risks of 2.3 (95% CI, 1.5-3.3) reported in a meta-analysis of hospital- and population-based studies.^3^ Fewer studies investigated the risk of IS after ICH by index hematoma location, and each had smaller samples than in our study.^3,7,8^ Our finding of an adjusted hazard ratio of 0.81 (95% CI, 0.60-1.10) for risk of subsequent IS after lobar vs nonlobar ICH is consistent with the pooled estimate of 0.8 (95% CI, 0.5-1.2) reported in a meta-analysis.^3^

Similar to another study,^3^ we found that lobar ICH location was associated with recurrent ICH, whereas comorbid AF (without reference to index hematoma location) was associated with a high risk of ischemic events but not recurrent ICH. We also found that risk estimates for those with previous occlusive vascular disease were higher for both recurrent ICH and IS and of a magnitude similar to those with AF. When considering both hematoma location and comorbid AF, we found that the risk of recurrent ICH was higher than the risk of IS only in patients with lobar ICH and no comorbid AF, as previously reported.^3^ Interestingly, we found that the highest relative risk of recurrent ICH was in patients with an index lobar hematoma in combination with a history of previous occlusive vascular disease, a composite risk factor^3^ that merits further study.

The timing of ICH recurrence has been reported in only a few studies.^5,22^ To our knowledge, only 1 previous study^3^ reported short-term ICH recurrence risk by index hematoma location in which the risk of recurrent ICH was highest in the first 90 days after a lobar ICH. Similarly, we observed a higher risk of recurrent ICH in the first year after a lobar index hematoma, particularly during the first 30 days. In contrast, we found that patients with nonlobar ICH were at increased risk of ischemic events (ie, IS and MI) in the first 30 days after ICH. Together, these findings may have potential clinical implications because they identify a group of vulnerable patients who might benefit from more targeted prevention efforts.^22^

Our finding of a higher short-term case fatality rate in survivors of ICH with recurrent ICH compared with those who had IS is consistent with a nationwide Danish study^5^ based exclusively on registry data. Our finding of greater gait impairment after recurrent ICH than IS further emphasizes the poorer prognosis after ICH.

We found that the risk of a second recurrent stroke (ie, a stroke occurring after the outcome stroke in the primary analysis) did not differ by lobar vs nonlobar index hematoma location and that the risk of recurrent IS was higher than for a second recurrent ICH, irrespective of baseline hematoma location (eTable 8 in Supplement 1). We regard this finding as hypothesis generating and requiring replication and exploration in future studies.

### Strengths and Limitations

Our study has several strengths. We used multiple sources to identify patients in the spontaneous ICH cohort at baseline, an approach that, combined with our inclusion of all hospitals in the catchment area, minimizes selection bias.^24^ The more selective approach we used when tracking events during follow-up (ie, not including patients exclusively diagnosed with ICH as outpatients) was supported by findings from a validation study.^14^ Our use of nationwide registries allowed us to track long-term medication use after ICH and to determine vital status with virtually no loss to follow-up apart from the low rate of emigration.^5^

Limitations of our study include our use of unverified registry diagnostic codes for MI. Myocardial infarction diagnosis codes, however, are reported to have a high positive predictive value,^16^ and it is unlikely that the positive predictive value for MI would vary by hematoma location. Although this study is one of the largest of its kind, it was underpowered to robustly assess the risk of MI or a second stroke during follow-up. Although our method of ascertaining hematoma location was based primarily on brain scan reports and discharge summaries, the approach is valid and compares favorably with masked systematic evaluation of hematoma location.^19^ We, however, lacked data on brain scan characteristics other than hematoma location, which could influence short-term mortality (eg, ICH volume) and stroke recurrence risk (eg, presence of cerebral microbleeds). Importantly, not all patients subsequently underwent magnetic resonance imaging or computed tomography angiography, and we can therefore not rule out a nonspontaneous ICH in some of these patients. We mitigated this potential source of misclassification by excluding patients younger than 50 years, the age group most likely to have a nonspontaneous parenchymal hemorrhage. Our secondary outcome concerning the ability to walk 3 months after recurrent stroke was based on hospital medical records and therefore liable to some degree of underestimation of recovery (eg, for patients who improved after discharge from acute care or rehabilitation units). This underestimation of recovery of gait may have been further accentuated by our choice of a 3-month window, which may be too short to assess outcomes after ICH.^25^ We had insufficient data on some potential confounders (eg, socioeconomic status and blood pressure measurements) and needed to use proxies for alcohol and smoking. We did not collect data on the study population’s ancestry because the population of Denmark (and, therefore, this cohort) is of mainly European ancestry; therefore, our results may not be generalizable to other populations.

## Conclusions

In this cohort study, lobar ICH was associated with a higher risk of MACEs than nonlobar ICH, and this higher risk was largely attributable to higher rates of recurrent ICH. Our novel observation that the risk of a second stroke after ICH did not differ by index hematoma location and that this risk was higher for IS than ICH merits further study.

## Notes

Supplement 1. **eMethods.** Supplemental Methods **eResults.** Supplemental Results
eReferences
**eTable 1.** Diagnosis and Procedure Codes for Study Covariates **eTable 2.** ICH Location Established According to the Study Method (I.E, Based on Brain Scan Reports and Discharge Summaries) Compared With Re-evaluation of Brain CT Scans According to CHARTS by Evaluators Blinded to Clinical Data **eTable 3.** Relative Rates (Unadjusted Hazard Ratios and Subdistribution Hazard Ratios) for Main Outcomes Stratified by First-Ever ICH Hematoma Location and by Select Comorbidities **eTable 4.** Relative Rates (Unadjusted Hazard Ratios and Subdistribution Hazard Ratios) for Main Outcomes Stratified by Hematoma Location Within Strata of Patients With or Without Atrial Fibrillation or Previous Occlusive Vascular Disease, Respectively **eTable 5.** Absolute Event Rates for Recurrent Intracerebral Hemorrhage, Ischemic Stroke, and Myocardial Infarctions on Days 0-30 After Onset of Index ICH and on Days 31 to End of Study Period **eTable 6.** Case-Fatality Rate and Functional Outcome of First Recurrent Stroke in Patients Followed Up After Their First-Ever Intracerebral Hemorrhage **eTable 7.** Absolute Event Rates for Main Outcomes Stratified by Location of Baseline ICH and Based on Individual Follow-up for Each Outcome With Censoring Only Contingent on Migration, Death, or End of Study Period (as Opposed to Main Analysis, Where First Occurrence of a Primary Outcome Was Also a Censoring Criterion) **eTable 8.** Absolute and Relative Event Rates for Second Recurrent Stroke by Hematoma Location of Index ICH **eTable 9.** Baseline Characteristics of Patients at Risk of a Second Ischemic Stroke During Follow-up Stratified by Hematoma Location of the Index ICH **eFigure 1.** Study Flowchart **eFigure 2.** Cumulative Incidence of Main Outcomes by ICH Location Taking Competing Risk of Death Into Account **eFigure 3.** Cumulative Incidence of Main Outcomes by ICH Location (Lobar vs Deep) in Patients With/Without Comorbid Atrial Fibrillation at Baseline **eFigure 4.** Cumulative Incidence of Main Outcomes by ICH Location (Lobar vs Deep) in Patients With/Without Previous Occlusive Vascular Disease at Baseline **eFigure 5.** Annual Incidence Rate (95% Confidence Intervals Indicated With Bars) of Main Outcomes for Lobar ICH and Nonlobar ICH

Supplement 2.
Data Sharing Statement
