# Supplementary material for: Major Cardiovascular Events After Spontaneous Intracerebral Hemorrhage by Hematoma Location
Source: JAMA Netw Open. 2023 Apr 5;6(4):e235882. doi: 10.1001/jamanetworkopen.2023.5882 (PMC10077102; doi:10.1001/jamanetworkopen.2023.5882)
Supplement: Supplement 1. — eMethods. Supplemental Methods eResults. Supplemental Results eReferences eTable 1. Diagnosis and Procedure Codes for Study Covariates eTable 2. ICH Location Established According to the Study Method (I.E, Based on Brain Scan Reports and Discharge Summaries) Compared With Re-evaluation of Brain CT Scans According to CHARTS by Evaluators Blinded to Clinical Data eTable 3. Relative Rates (Unadjusted Hazard Ratios and Subdistribution Hazard Ratios) for Main Outcomes Stratified by First-Ever ICH Hematoma Location and by Select Comorbidities eTable 4. Relative Rates (Unadjusted Hazard Ratios and Subdistribution Hazard Ratios) for Main Outcomes Stratified by Hematoma Location Within Strata of Patients With or Without Atrial Fibrillation or Previous Occlusive Vascular Disease, Respectively eTable 5. Absolute Event Rates for Recurrent Intracerebral Hemorrhage, Ischemic Stroke, and Myocardial Infarctions on Days 0-30 After Onset of Index ICH and on Days 31 to End of Study Period eTable 6. Case-Fatality Rate and Functional Outcome of First Recurrent Stroke in Patients Followed Up After Their First-Ever Intracerebral Hemorrhage eTable 7. Absolute Event Rates for Main Outcomes Stratified by Location of Baseline ICH and Based on Individual Follow-up for Each Outcome With Censoring Only Contingent on Migration, Death, or End of Study Period (as Opposed to Main Analysis, Where First Occurrence of a Primary Outcome Was Also a Censoring Criterion) eTable 8. Absolute and Relative Event Rates for Second Recurrent Stroke by Hematoma Location of Index ICH eTable 9. Baseline Characteristics of Patients at Risk of a Second Ischemic Stroke During Follow-up Stratified by Hematoma Location of the Index ICH eFigure 1. Study Flowchart eFigure 2. Cumulative Incidence of Main Outcomes by ICH Location Taking Competing Risk of Death Into Account eFigure 3. Cumulative Incidence of Main Outcomes by ICH Location (Lobar vs Deep) in Patients With/Without Comorbid Atrial Fibrillation at Baseline eFigure [file jamanetwopen-e235882-s001.pdf]

Supplementary Online Content

Boe NJ, Hald SM, Jensen MM, et al. Major cardiovascular events after spontaneous intracerebral hemorrhage by hematoma location. *JAMA Netw Open*. 2023;6(4):e235882. doi:10.1001/jamanetworkopen.2023.5882

eMethods. Supplemental Methods
eResults. Supplemental Results
eReferences
eTable 1. Diagnosis and Procedure Codes for Study Covariates
eTable 2. ICH Location Established According to the Study Method (I.E, Based on Brain Scan Reports and Discharge Summaries) Compared With Re-evaluation of Brain CT Scans According to CHARTS by Evaluators Blinded to Clinical Data
eTable 3. Relative Rates (Unadjusted Hazard Ratios and Subdistribution Hazard Ratios) for Main Outcomes Stratified by First-Ever ICH Hematoma Location and by Select Comorbidities
eTable 4. Relative Rates (Unadjusted Hazard Ratios and Subdistribution Hazard Ratios) for Main Outcomes Stratified by Hematoma Location Within Strata of Patients With or Without Atrial Fibrillation or Previous Occlusive Vascular Disease, Respectively
eTable 5. Absolute Event Rates for Recurrent Intracerebral Hemorrhage, Ischemic Stroke, and Myocardial Infarctions on Days 0-30 After Onset of Index ICH and on Days 31 to End of Study Period
eTable 6. Case-Fatality Rate and Functional Outcome of First Recurrent Stroke in Patients Followed Up After Their First-Ever Intracerebral Hemorrhage
eTable 7. Absolute Event Rates for Main Outcomes Stratified by Location of Baseline ICH and Based on Individual Follow-up for Each Outcome With Censoring Only Contingent on Migration, Death, or End of Study Period (as Opposed to Main Analysis, Where First Occurrence of a Primary Outcome Was Also a Censoring Criterion)
eTable 8. Absolute and Relative Event Rates for Second Recurrent Stroke by Hematoma Location of Index ICH
eTable 9. Baseline Characteristics of Patients at Risk of a Second Ischemic Stroke During Follow-up Stratified by Hematoma Location of the Index ICH
eFigure 1. Study Flowchart
eFigure 2. Cumulative Incidence of Main Outcomes by ICH Location Taking Competing Risk of Death Into Account
eFigure 3. Cumulative Incidence of Main Outcomes by ICH Location (Lobar vs Deep) in Patients With/Without Comorbid Atrial Fibrillation at Baseline
eFigure 4. Cumulative Incidence of Main Outcomes by ICH Location (Lobar vs Deep) in Patients With/Without Previous Occlusive Vascular Disease at Baseline
eFigure 5. Annual Incidence Rate (95% Confidence Intervals Indicated With Bars) of Main Outcomes for Lobar ICH and Nonlobar ICH

This supplementary material has been provided by the authors to give readers additional information about their work.

© 2023 Boe NJ initials et al. *JAMA Network Open.*

eMethods. Supplement Methods.

eSetting

Acute stroke is regarded as a medical emergency and suspected cases are offered rapid evaluation with easy access to brain imaging. In Denmark, patients who have a stroke are admitted to stroke units. The Region of Southern Denmark (RSD; population 1.2 million) includes four neurological departments with stroke units and one neurosurgery department. During the study period, brain computed tomography (CT) was the first-line imaging investigation for suspected acute stroke in hospitals in RSD.

Identification and verification of first-ever ICH cohort

As previously described,[1] we identified a cohort of first-ever intracerebral hemorrhage (ICH) in Southern Denmark through two nationwide Danish registries, the Danish Stroke Registry (Stroke Registry) and the Danish National Patient Registry (Patient Registry). We retrieved data on patients residing in Southern Denmark who were recorded under International Classification of Diseases version 10 codes for ICH in the Stroke Registry (for the period January 1, 2003 – the year the registry became operative – to December 31, 2018), or the Patient Registry (primary or secondary code position, admission, emergency room, or outpatient contacts for the period January 1, 2007 to December 31, 2018). To minimize misclassification of prevalent ICH cases classified as first-ever ICH, we excluded patients recorded in either registry with ICH diagnosis codes predating the study period. For a total of 4,621 patients with hospital contacts with a first-ever ICH code recorded in either registry during the study period, we retrieved primarily discharge summaries and brain scan reports using a previously validated method to ascertain the spontaneous nature of ICH and hematoma location.[2] We could not trace medical records in 191 (4%) of cases, mainly due to paper-based medical records no longer being available in the archives of some hospitals.[1] Other reasons for exclusion of patients were: ICH diagnosis verified but not spontaneous (n=816), intracranial hemorrhage other than ICH (n=469), ischemic stroke or TIA (n=122), other coding errors (n=194), and spontaneous ICH but prevalent (i.e., first-ever ICH onset before study period) (n=10).[3] A total of 2,819 patients with verified first ever spontaneous ICH were eligible for this study. After exclusion of a further 500 patients for reasons outlined in eFigure 1, the final sample for the main analysis comprised 1,034 lobar and 1,255 non-lobar patients with first-ever ICH.

Although we cannot document the exact completeness of our data regarding first-ever ICH we feel confident that it is high. However, some caveats must be kept in mind. We were not able to identify patients who died with ICH before reaching a hospital. As the rate of autopsy in Denmark is low,[4] supplementing our data with information from the Cause-of-Death Registry would most likely not improve our estimates to any measurable degree.[1] Also, we did not have access to information on patients suffering an ICH that were not referred to hospital for evaluation. However, given the structure of the Danish health system, including the availability of fast-track evaluation of potential stroke, we believe that the magnitude of this selection bias is relatively small.[1]

Identification and verification of recurrent stroke and spontaneous intracranial hemorrhages other than ICH

To track recurrent ICH, ischemic stroke (IS) and intracranial hemorrhages other than ICH occurring during follow-up of the inception cohort, we interrogated the Patient Registry and the Stroke Registry. In the Patient Registry, we identified in-hospital contacts with International Classification of Disorders version 10 (ICD-10) discharge diagnosis codes (primary or non-primary coding position) corresponding to ICH (ICD-10 code: I61), IS (I63, I66), stroke unspecified (I64), some less specific codes that might have been used to identify stroke (I67.8, G46), transient ischemic attack (G45.9),

© 2023 Boe NJ initials et al. *JAMA Network Open.*

subarachnoid hemorrhage (SAH) (I60), subdural hematoma (SDH) (I62.0, S06.5), non-traumatic epidural hematoma (I621) and intracranial hematoma unspecified (I62.9). From the Stroke Registry we retrieved admissions coded as ICH or IS. For all admissions concerned, whether in Patient Registry or Stroke Registry, we traced medical records (including reports of initial and control brain scans). We could not trace medical records for less than 2% of interrogated hospital contacts. Rather than accept the diagnosis code at face value, we chose to exclude these contacts from further consideration.

Based on information from retrieved medical records, 4 study physicians, supervised by a neurologist with special interest in stroke (DG), classified symptomatic spontaneous (non-traumatic) intracranial events after index ICH into re-ICH, IS, TIA, and intracranial extra axial hemorrhages (ICrExH) (i.e., SAH, SDH, or epidural). In cases of doubt regarding imaging issues, original scans were reviewed.

Information on episodes of TIA was not included in this study.

Potential confounders considered for inclusion in multivariable analyses

The following were considered for inclusion as potential confounders: hypertension, atrial fibrillation (AF), previous IS, myocardial infarction (MI), peripheral arterial disease, diabetes, chronic obstructive pulmonary disease (as a marker of smoking), diagnoses indicative of high alcohol use, current use of medications [separate covariates for each of the following drug classes: platelet antiaggregants (low-dose aspirin or clopidogrel), anticoagulants (direct oral anticoagulants (DOACs) or vitamin K antagonist (VKA)), statins, and antihypertensives]. These variables were chosen on the basis of subject matter knowledge. The above potential confounders were classified at baseline, i.e., based on information recorded in registries in a fixed time window of the 15 years leading up to and including the start of follow-up (start date). Use of antithrombotics, statins, and antihypertensive medications was also tracked during follow-up in a sensitivity analysis (see below).

Classification of exposure to medications

We retrieved data on prescriptions dispensed to cohort members from the Danish National Prescription Registry.[5] Baseline medication exposure was classified into current use (latest prescription supply before start date lasted until start date, or ended within the 30 previous days), past use (supply ended 31-365 days before index date), and non-use (supply ended >365 days before index date or no prescription for the drug recorded in 15-year time-window). For the purposes of a sensitivity analysis, we also created time-dependent variables to capture varying exposure to platelet antiaggregants, anticoagulants, statins, or antihypertensive drugs (the latter as part of confounder control) during follow-up. Patients entered the follow-up with status of 'current use', 'past use', or 'non-use' classified as described above. A patient's status with regard to use of e.g., platelet antiaggregant drugs, could change during follow-up in a time dependent manner as further prescriptions were presented. For example, if no further prescriptions were presented 31-365 days after platelet antiaggregant drug supply run out, current use status changed to past use (or non-use, if supply had run out for >365 days). Conversely, past (or non-use) status was changed to current use if a prescription for the drug in question was presented during follow-up.

Validation of study method used to classify hematoma location

We previously investigated the validity of classifying location of the index ICH based exclusively on retrieved brain scan reports and discharge summaries (henceforth referred to as simple method) in a small sample of patients.[2] To further explore the validity of the simple method, we compared it to results achieved when using an internationally recognized

rating instrument, CHARTS[6] in a large subsample of patients (all patients aged 55+ years with an index ICH in 2015-2018). For the purposes of this analysis, we included all index ICH patients fulfilling the age and time-period criteria with a single hematoma, irrespective of location. For each of these patients the first brain CT performed after the onset of the index ICH was evaluated blinded to all clinical data (including previous radiological reports) by two radiology trainees (approximately 1 year of experience), supervised by an experienced radiologist with a special interest in neuroradiology. The simple method allows classification into 'lobar', 'non-lobar', 'isolated intraventricular hemorrhage', 'large unclassifiable ICH', and 'unclassifiable due to missing information'. The CHARTS classifications we employed were 'lobar', 'probable lobar', 'deep', 'probable deep', and 'holohemispheric'. We cross tabulated the results of the two methods and calculated kappa statistics for agreement; we also calculated positive predictive values and corresponding 95% CIs for lobar and non-lobar location according to the simple method using the CHARTS result as gold standard.

eResults. Supplement Results.

Subanalyses

Absolute event rates per 100 person-years for recurrent ICH, IS, and MI were higher in the day 0-30 period than in the day ≥31 period of follow-up (**eTable 5**). The type of event in the first 30-days after ICH varied by index hematoma location (i.e., the lobar cohort had a higher risk of early recurrent ICH whereas the non-lobar cohort was at higher risk of early ischemic events (i.e., IS or MI) (**eTable 5**). Annual IRs for the main outcomes were higher in the first year for MACE and recurrent ICH in the lobar cohort compared with the non-lobar cohort (**eFigure 5**).

The case fatality rate was higher and percentage of patients able to walk unaided was lower after recurrent ICH than after IS for patients with non-lobar ICH; this was also true for the ability to walk unaided in the lobar cohort (**eTable 6**). Compared with the lobar cohort, patients in the non-lobar cohort with recurrent ICH had a higher case fatality on day 1 and day 7.

Absolute rates independent of other outcomes produced results similar to main analyses (**eTable 7**). We used this approach for calculating rates independent of other outcomes in analyses of second recurrent strokes. The IR per 100 person-years was similar between lobar and non-lobar cohorts for second ICH (lobar: 3.49 [95%CI, 1.57-7.77] vs. non-lobar: 3.53 [95%CI, 0.88-14.13]) and for second IS (lobar: 6.61 [95%CI, 2.48-17.60] vs. non-lobar: 4.71 [95%CI, 1.77-12.56])) (Table 4). Compared to the overall cohorts (Table 1), baseline characteristics of patients with a second IS during follow-up had a somewhat higher burden of risk factors (e.g., higher prevalence of men [lobar 61.8%; non-lobar 55.8%] and previous ischemic stroke [lobar 17.6%; non-lobar 16.7%], whereas baseline comorbid AF was similar (lobar 20.6%; non-lobar 25.0%; **eTable 8**)

Sensitivity analyses

Sensitivity analysis of the relative risk for main outcomes after follow-up of the lobar versus non-lobar cohort with additional adjustment for use of medication (antihypertensives, platelets antiaggregants, oral anticoagulants, and statins) during follow-up produced results similar to the main analysis (e.g., aHR [95%CI] lobar vs. non-lobar (reference), main vs. supplementary analysis: recurrent ICH, 2.63 [1.97-3.49] vs. 2.62 (1.97-3.49); IS, 0.81 (0.60-1.10) vs. 0.83 [0.61-1.12]).

© 2023 Boe NJ initials et al. *JAMA Network Open.*

eReferences

1. Hald S, Sloth C, Agger M, et al. The validity of intracerebral hemorrhage diagnoses in the Danish Patient Registry and the Danish Stroke Registry. *Clin Epidemiol*. 2020;12:1313-1326. doi:https://doi.org/10.2147/CLEP.S267583

2. Hald SM, Kring Sloth C, Hey SM, et al. Intracerebral hemorrhage: positive predictive value of diagnosis codes in two nationwide Danish registries. *Clin Epidemiol*. 2018;10:941-948. doi:10.2147/CLEP.S167576

3. Boe NJ, Hald SM, Jensen MM, et al. Association Between Statin Use and Intracerebral Hemorrhage Location: A Nested Case-Control Registry Study. *Neurology*. Published online December 7, 2022:10.1212/WNL.0000000000201664. doi:10.1212/WNL.0000000000201664

4. Petri CN. Decrease in the frequency of autopsies in Denmark after the introduction of a new autopsy act. *Qual Assur Health Care Off J Int Soc Qual Assur Health Care*. 1993;5(4):315-318. doi:10.1093/intqhc/5.4.315

5. Pottegård A, Schmidt SAJ, Wallach-Kildemoes H, Sørensen HT, Hallas J, Schmidt M. Data Resource Profile: The Danish National Prescription Registry. *Int J Epidemiol*. Published online October 27, 2016. doi:10.1093/ije/dyw213

6. Charidimou A, Schmitt A, Wilson D, et al. The Cerebral Haemorrhage Anatomical RaTing inStrument (CHARTS): Development and assessment of reliability. *J Neurol Sci*. 2017;372:178-183. doi:10.1016/j.jns.2016.11.021

© 2023 Boe NJ initials et al. *JAMA Network Open.*

eTable 1. Diagnosis and procedure codes for study covariates

	Diagnosis Codes (ICD-10) or Procedure codes	Drug (ATC) codes
**Outcomes**[1]		
**1**. Intracerebral hemorrhage (ICH)	Not applicable: Verified diagnoses only	
**2**. Ischemic stroke	Not applicable: Verified diagnoses only	
**3**. Intracranial hemorrhage other than ICH (ICrExH)	Not applicable: Verified diagnoses only	
**4**. Myocardial infarct	I21, I22, I23	
**5**. MACE (major adverse cardiovascular event)		
5a. Stroke	ICH or ischemic stroke as defined above [stroke of uncertain type not included as all strokes were verified]	
5b. Myocardial infarction	As defined above	
5c. Systemic embolism	I74	
5d. Vascular death - Death within 30 days of a hospital discharge for one or more of the following events:		
5d1. Any of main outcomes (stroke, ICrExH, MI, or systemic embolism)	As defined above	
Any of following non-stroke events		
5d2. Revascularization procedures		
5d2a. Thrombolysis (any organ)	BOHA1 (DNPR, sksube)	
5d2b. Percutaneous coronary intervention (PCI) [includes stenting]	KFNG, KFNF (DNPR, sksube)	
5d2c. Coronary artery bypass grafting (CABG)	KFNA, KFNB, KFNC, KFND, KFNE, KFNH20 (DNPR, sksube)	
5d2d. Carotid artery procedures (includes all arteries derived from aortic arch and its branches)	KPAF (endarterectomy), KPAE (thrombectomy) (DNPR, sksube)	
5d2e. Visceral artery, thrombectomy/embolectomy (mesenteric, renal, visceral)	KPCE, KPCF (DNPR, sksube)	
5d2f. Lower limb revascularization (thrombectomy/embolectomy, stents, bypass surgery etc)	KPDE, KPDF, KPDH, KPDN, KPDP, KPDQ, KPEE, KPEF, KPEH, KPEN, KPEP, KPEQ, KPFE, KPFH, KPFN, KPFP, KPFQ, KPDU74, KPDU84 (DNPR, sksube)	
5d3. Mesenteric ischaemia	K550E K550H (DNPR, diag)	
5d4. Sudden cardiac death	I461 (DNPR, diag)	
5d5. Venous thromboembolism (DVT, or pulmonary embolism)	I26, I801, I802, I803, I808, I809 (DNPR, diag)	
5d6. Extracranial hemorrhage		
5d7a. Gastrointestinal bleed (upper, lower, or unspecified)	K228F, K250, K252, K254, K256, K260, K262, K638B, K264, K266, K270, K272, K274, K276, K280, K282, K284, K286, K290, K298A, K625, K638B, K638C, K290-K292, K920, K921, K922 (DNPR, diag)	
5d7b. Esophageal varicose vein hemorrhage or esophageal hemorrhage	I850, I864A, K228F (DNPR, diag)	
5d7c. Hematuria	N02, R31 (DNPR, diag)	

© 2023 Boe NJ initials et al. *JAMA Network Open.*

5d7d. Hemopericardium	I312 (DNPR, diag)	
5d7e. Peritoneal hemorrhage	K661(DNPR, diag)	
5d7f. Hemothorax	J942 (DNPR, diag)	
5d7g. Hemorrhage in bile duct or pancreas	K838F, K868G (DNPR, diag)	
5d7h. Hemorrhage in spinal cord	G951A (DNPR, diag)	
5d7i. Acute bleeding anemia	D62 (DNPR, diag)	
**Covariates – disorders**[2]		
**Based on diagnoses/drug use**		
Hypertension	I10-I15	C03A, C08CA, C08DB01, C09A, C09B, C09C, C09D
Atrial fibrillation	I48	
Occlusive vascular disease (ischemic stroke, myocardial infarction, or peripheral arterial disease)	Ischemic stroke/Myocardial infarction: as defined under Outcomes<br>Peripheral arterial disease: as defined below	
Ischemic stroke	I63 or I64 in Danish Stroke Registry	
Myocardial infarction	As defined under Outcomes	
Peripheral arterial disease	As defined below	
Diabetes	E10-E14	A10
Chronic obstructive pulmonary disorder	J42, J42, J44	R03 [in subjects aged 45+ years]
High alcohol intake (disorders/events or drug use indicative of alcohol abuse)	E244, F10, G312, G621, G721, I426, K292, K70, K860, T510, T519, Z502, Z714, Z721,	N07BB
Peripheral arterial disease (PAD)	I702, I739	
Chronic kidney diseases	N18 (excluding N181), N19, Z992, Z940	
**Covariates – use of drugs (separate covariates for the four drug classes)**		
Platelet antiaggregant s (low-dose aspirin [alone or in combination with dipyridamole], or clopidogrel)		B01AC06 – acetylsalicylic acid (75 mg, 100 mg, or 150 mg per tablet)<br>B01AC30 - acetylsalicylic acid (50 mg per tablet) in combination with dipyridamole<br>B01AC04 (75 mg per tablet)
Oral anticoagulants		B01AA – vitamin K antagonist<br>B01AE07, B01AF01, B01AF02, B01AF03 – direct oral anticoagulants
Statins		C10AA
Drugs with antihypertensive effects[3]<br><br>[thiazides and other non-loop diuretics, loop-diuretics, beta-blockers, calcium channel blockers, ACE-inhibitor and angiotensin II receptor blockers (plain or in combinations]		C03A, C03D, C03E, C03C, C07, CO8, C09A, C09B, C09C, C09D

© 2023 Boe NJ initials et al. *JAMA Network Open.*

[1]Verified diagnoses for stroke outcomes and intracranial extraaxial hemorrhages; for other outcomes (e.g., myocardial infarction) primary diagnosis codes in inpatient encounters only.

[2]Primary or secondary diagnosis position codes in inpatient or outpatient data.

[3]Indication for drug use not available to us and some of these drugs may have been used for indications other than hypertension. Note that ‘ever use’ of some of these drugs was included in definition of hypertension covariate (i.e., C03A, C08CA, C08DB01, C09A, C09B, C09C, C09D).

© 2023 Boe NJ initials et al. *JAMA Network Open.*

eTable 2. ICH location established according to the study method (i.e, based on brain scan reports and discharge summaries) compared with re-evaluation of brain CT scans according to CHARTS by evaluators blinded to clinical data. Numbers where evaluation methods are in agreement in bold.

		**Evaluation according to study method**[1]				
		Lobar	Deep	Large un-classifiable	Isolated intraventricular hemorrhage	Unclassifiable, missing information
**CHARTS evaluation**	Lobar	**335**	12	22	NR[2]	NR[2]
	Probable lobar	**19**	7	29	NR[2]	0
	Deep	19	**441**	34	NR[2]	NR[2]
	Probable deep	11	**23**	17	0	0
	Holohemispheric	NR[1]	0	**15**	0	0

[1]Only patients with a single hematoma and index ICH event in 2015-2018 included (n=1,001); 58 with multiple concurrent ICHs not included in above comparison.
[2]Not reported (n≤5) to preserve anonymity.

© 2023 Boe NJ initials et al. *JAMA Network Open.*

eTable 3. Relative rates (unadjusted hazard ratios and subdistribution hazard ratios) for main outcomes stratified by first-ever ICH hematoma location and by select comorbidities.

	**First-ever ICH hematoma location**[1]		**Atrial fibrillation**[2]		**Previous occlusive disorder**[2,3]	
	Lobar (n=1,034)	Non-lobar (n=1,255)	Yes (n=489)	No (n=1,800)	Yes (n=512)	No (n=1,777)
**Recurrent ICH**						
Relative rate – uHR[4] (95% CI)	2.98 (2.00; 4.43)	1 (reference)	0.59 (0.32; 1.10)	1 (reference)	1.20 (0.76; 1.90)	1 (reference)
Relative rate – sdHR[5] (95% CI)	2.58 (1.94; 3.43)	1 (reference)	0.79 (0.67; 0.92)	1 (reference)	1.61 (1.27; 2.05)	1 (reference)
**Ischemic stroke**						
Relative rate – uHR[4] (95% CI)	0.82 (0.52; 1.28)	1 (reference)	1.74 (1.04; 2.91)	1 (reference)	1.70 (1.03; 2.79)	1 (reference)
Relative rate – sdHR[5] (95% CI)	0.79 (0.58; 1.08)	1 (reference)	1.46 (1.18; 1.79)	1 (reference)	1.50 (1.12; 2.00)	1 (reference)
**Myocardial infarction**						
Relative rate – uHR[4] (95% CI)	0.65 (0.29; 1.45)	1 (reference)	2.37 (1.04; 5.43)	1 (reference)	2.03 (0.89; 4.64)	1 (reference)
Relative rate – sdHR[5] (95% CI)	0.63 (0.36; 1.10)	1 (reference)	2.48 (1.89; 3.26)	1 (reference)	0.98 (0.55; 1.74)	1 (reference)
**MACE**[6]						
Relative rate – uHR[4] (95% CI)	1.29 (1.07; 1.56)	1 (reference)	1.50 (1.20; 1.87)	1 (reference)	1.39 (1.12; 1.74)	1 (reference)
Relative rate – sdHR[5] (95% CI)	1.25 (1.09; 1.42)	1 (reference)	2.48 (2.31; 2.65)	1 (reference)	1.33 (1.17; 1.51)	1 (reference)

© 2023 Boe NJ initials et al. *JAMA Network Open.*

eTable 3 – continued

	Diabetes[2]	
	Yes (n=305)	No (n=1,984)
**Recurrent ICH**		
Relative rate – uHR[4] (95% CI)	0.40 (0.17; 0.90)	1 (reference)
Relative rate – sdHR[5] (95% CI)	0.99 (0.78; 1.26)	1 (reference)
**Ischemic stroke**		
Relative rate – uHR[4] (95% CI)	0.89 (0.44; 1.78)	1 (reference)
Relative rate – sdHR[5] (95% CI)	0.55 (0.38; 0.79)	1 (reference)
**Myocardial infarction**		
Relative rate – uHR[4] (95% CI)	NE[7]	1 (reference)
Relative rate – sdHR[5] (95% CI)	NE[7]	1 (reference)
**MACE[6]**		
Relative rate – uHR[4] (95% CI)	0.98 (0.73; 1.30)	1 (reference)
Relative rate – sdHR[5] (95% CI)	0.98 (0.86; 1.12)	1 (reference)

[1]Location of hematoma on brain scan of first-ever intracerebral hemorrhage.

[2]Classified based on information at baseline.

[3]Medical history of ischemic stroke, myocardial infarction, or peripheral arterial disease at baseline.

[4]Unadjusted hazard ratio.

[5]Hazard ratio using Fine-Gray sub-distribution with all-cause death as competing event and adjusted for sex, age (<75 (ref); 75-84; 85+ years), hypertension, atrial fibrillation, previous ischemic stroke, myocardial infarction, peripheral arterial disease, diabetes, chronic obstructive pulmonary disease (as a marker of smoking), diagnoses indicative of high alcohol use, use of medications (separate covariates for each of the following drug classes: platelet antiaggregants (low-dose aspirin or clopidogrel), anticoagulants (direct oral anticoagulants or vitamin K antagonist), antihypertensives, and statins).

[6]Major adverse cardiovascular event defined as stroke (ICH, or ischemic stroke), myocardial infarction, systemic embolism, or vascular death.

[7]Not estimated due to sparse events.

© 2023 Boe NJ initials et al. *JAMA Network Open.*

eTable 4. Relative rates (unadjusted hazard ratios and subdistribution hazard ratios) for main outcomes stratified by hematoma location within strata of patients with or without atrial fibrillation or previous occlusive vascular disease, respectively.

	**Atrial fibrillation**[1]			
	**Yes**		**No**	
	**Lobar**[3] (n=216)	**Non-lobar**[3] (n=273)	**Lobar**[3] (n=818)	**Non-lobar**[3] (n=982)
**Recurrent ICH**				
Relative rate – uHR[4] (95% CI)	1.26 (0.38; 4.18)	1 (reference)	3.30 (2.16; 5.04)	1 (reference)
Relative rate – sdHR[5] (95% CI)	1.22 (0.53; 2.82)	1 (reference)	2.86 (2.11; 3.88)	1 (reference)
**Ischemic stroke**				
Relative rate – uHR[4] (95% CI)	0.53 (0.20; 1.40)	1 (reference)	0.92 (0.55; 1.53)	1 (reference)
Relative rate – sdHR[5] (95% CI)	0.51 (0.26; 0.98)	1 (reference)	0.90 (0.63; 1.27)	1 (reference)
**Myocardial infarction**				
Relative rate – uHR[4] (95% CI)	0.63 (0.15; 2.66)	1 (reference)	0.63 (0.24; 1.65)	1 (reference)
Relative rate – sdHR[5] (95% CI)	NE[6]	1 (reference)	0.60 (0.31; 1.19)	1 (reference)
**MACE**[8]				
Relative rate – uHR[4] (95% CI)	0.72 (0.48; 1.07)	1 (reference)	1.52 (1.23; 1.88)	1 (reference)
Relative rate – sdHR[5] (95% CI)	0.76 (0.58; 1.01)	1 (reference)	1.44 (1.24; 1.67)	1 (reference)
	**Previous occlusive vascular disease**[1,2]			
	**Yes**		**No**	
	**Lobar**[3] (n=218)	**Non-lobar**[3] (n=294)	**Lobar**[3] (n=816)	**Non-lobar**[3] (n=961)
**Recurrent ICH**				
Relative rate – uHR[4] (95% CI)	5.93 (2.30; 15.27)	1 (reference)	2.58 (1.66; 4.01)	1 (reference)
Relative rate – sdHR[5] (95% CI)	5.15 (2.53; 10.49)	1 (reference)	2.20 (1.61; 3.00)	1 (reference)
**Ischemic stroke**				
Relative rate – uHR[4] (95% CI)	1.31 (0.54; 3.20)	1 (reference)	0.76 (0.45; 1.27)	1 (reference)
Relative rate – sdHR[5] (95% CI)	1.20 (0.66; 2.17)	1 (reference)	0.71 (0.50; 1.02)	1 (reference)
**Myocardial infarction**				
Relative rate – uHR[4] (95% CI)	NR[7]	1 (reference)	0.70 (0.27; 1.77)	1 (reference)
Relative rate – sdHR[5] (95% CI)	0.51 (0.17; 1.51)	1 (reference)	0.72 (0.37; 1.39)	1 (reference)
**MACE**[8]				
Relative rate – uHR[4] (95% CI)	1.55 (1.05; 2.29)	1 (reference)	1.26 (1.02; 1.56)	1 (reference)
Relative rate – sdHR[5] (95% CI)	1.46 (1.11; 1.91)	1 (reference)	1.20 (1.03; 1.40)	1 (reference)

[1]Classified based on information at baseline.

[2]Medical history of ischemic stroke, myocardial infarction or peripheral arterial disease at baseline.

[3]Location of hematoma on brain scan of first-ever intracerebral hemorrhage.

[4]Unadjusted hazard ratio.

[5]Hazard ratio using Fine-Gray sub-distribution with all-cause death as competing event and adjusted for sex, age (<75 (ref); 75-84; 85+ years), hypertension, atrial fibrillation, previous ischemic stroke, myocardial infarction, peripheral arterial disease, diabetes, chronic obstructive pulmonary disease (as a marker of smoking), diagnoses indicative of high alcohol use, use of medications (separate covariates for each of the following drug classes: platelet antiaggregants (low-dose aspirin or clopidogrel), anticoagulants (direct oral anticoagulants or vitamin K antagonist), antihypertensives, and statins).

[6]Not estimated due to sparse events.

[7]Not reported to preserve anonymity in view of small cell counts.

[8]Major adverse cardiovascular event defined as stroke (ICH, or ischemic stroke), myocardial infarction, systemic embolism, or vascular death.

© 2023 Boe NJ initials et al. *JAMA Network Open.*

eTable 5. Absolute event rates for recurrent intracerebral hemorrhage, ischemic stroke, and myocardial infarctions on days 0-30 after onset of index ICH and on days 31 to end of study period. Censoring criteria as in main analysis.

	Event rate per 100 person-years (95% CI)							
	**Recurrent ICH**			**Ischemic stroke**			**Myocardial infarction**	
	**Day 0-30**	**Day ≥ 31**		**Day 0-30**	**Day ≥ 31**		**Day 0-30**	**Day ≥ 31**
All	3.58 (1.49; 8.60)	2.28 (1.89; 2.75)		2.86 (1.08; 7.63)	1.60 (1.28; 2.00)		1.43 (0.36; 5.73)	0.52 (0.35; 0.77)
Hematoma location[1]								
Lobar	7.86 (3.27; 18.89)	3.62 (2.88; 4.54)		No events	1.50 (1.05; 2.13)		No events	0.43 (0.23; 0.83)
Non-lobar	No events	1.28 (0.92; 1.78)		5.26 (1.97; 14.01)	1.68 (1.26; 2.24)		2.63 (0.66; 10.52)	0.58 (0.36; 0.95)

ICH: Intracerebral hemorrhage.

Small number of events in several strata and therefore only reported as rates to preserve anonymity.

[1]First-ever intracerebral hemorrhage hematoma location.

© 2023 Boe NJ initials et al. *JAMA Network Open.*

eTable 6. Case-fatality rate and functional outcome of first recurrent stroke in patients followed up after their first-ever intracerebral hemorrhage.

**Hematoma location of index ICH**	**Type of stroke occurring during follow-up**					***P-values***[1]		
**Lobar**	**Recurrent-ICH (n=80)**		**IS (n=31)**			**Recurrent ICH vs IS among patients with lobar index ICH**	**Recurrent ICH – lobar index ICH vs non-lobar index ICH**	**IS – lobar index ICH vs non-lobar index ICH**
	**Number**	**% (95% CI)**	**Number**	**% (95% CI)**				
**Case fatality rate**[2]								
Day 1	<5	NR	<5	NR		0.68	0.01	0.73
Day 7	12	15.0 (8.0; 24.7)	<5	NR		0.22	0.04	0.30
Day 30	22	27.5 (18.1; 38.6)	<5	NR		0.04	0.46	0.30
**Walked unaided after recurrent stroke**[3]	33	41.3 (30.4; 52.8)	18	58.1 (39.1; 75.5)		0.02	0.36	0.25
**Non-lobar**	**Recurrent-ICH (n=35)**		**IS (n=50)**			**Recurrent ICH vs IS Among patients with non-lobar index ICH**		
	**Number**	**% (95% CI)**	**Number**	**% (95% CI)**				
**Case fatality rate**[2]								
Day 1	7	20.0 (8.4; 36.9)	<5	NR		0.005	NA	NA
Day 7	11	31.4 (16.9; 49.3)	<5	NR		<0.001	NA	NA
Day 30	12	34.3 (19.1; 52.2)	9	18.0 (8.6; 31.4)		0.08	NA	NA
**Walked unaided after recurrent stroke**[3]	10	28.6 (14.6; 46.3)	23	46.0 (31.8; 60.7)		0.02	NA	NA

Abbreviations: ICH: intracerebral hemorrhage; IS: ischemic stroke; NA: Not applicable; NR: not reported (to preserve anonymity).

[1]Chi-squared test.

[2]Percentage of patients who died within 1, 7, and 30 days of admission for stroke. Includes all deaths (i.e. in- and out of hospital). Vital status retrieved from Danish Civil Registration System.

[3]Defined as being able to walk without support from other people, i.e., use of cane, crutch, or Zimmer frame allowed. Only patients who could walk unaided after index ICH and before recurrent stroke included. Classified based on all available information in medical records from departments of neurology, neurosurgery, or rehabilitation up to 3 months after onset of recurrent stroke. Less than 5 patients with missing information on ability to walk unaided (exact numbers not reported to preserve anonymity).

© 2023 Boe NJ initials et al. *JAMA Network Open.*

eTable 7. Absolute event rates for main outcomes stratified by location of baseline ICH and based on individual follow-up for each outcome with censoring only contingent on migration, death, or end of study period (as opposed to main analysis, where first occurrence of a primary outcome was also a censoring criterion).

Event during follow-up								
	Recurrent ICH			Ischemic stroke			Myocardial infarction	
	No. of events / person-years	Event rate per 100 pyrs (95% CI)		No. of events / person-years	Event rate per 100 pyrs (95% CI)		No. of events / person-years	Event rate per 100 pyrs (95% CI)
All (n=2,289)	122/5,169	2.36 (1.98; 2.82)		86/5,251	1.64 (1.33; 2.02)		28/5,344	0.52 (0.36; 0.76)
Index ICH location								
Lobar (n=1,034)	85/2,222	3.82 (3.09; 4.73)		34/2,330	1.46 (1.04; 2.04)		10/2,378	0.42 (0.23; 0.78)
Non-lobar (n=1,255)	37/2,947	1.26 (0.91; 1.73)		52/2,921	1.78 (1.36; 2.34)		18/2,966	0.61 (0.38; 0.96)

© 2023 Boe NJ initials et al. *JAMA Network Open.*

eTable 8. Absolute stroke event rates for second recurrent stroke by hematoma location of index ICH.

**All**	**Intracerebral hemorrhage[a]**					**Ischemic stroke[a]**			
Event no. during follow-up	No. at risk[b]	Events	Person-years	Event rate per 100 pyrs (95% CI)		No. at risk[b]	Events	Person-years	Event rate per 100 pyrs (95% CI)
First[c] recurrent stroke	2,289	122	5,169	2.36 (1.98-2.82)		2,289	86	5,251	1.64 (1.33-2.02)
Second recurrent stroke	122	NR[d]	NR[d]	3.50 (1.75-7.00)		86	NR[d]	NR[d]	5.50 (2.75-11.00)
**Lobar**									
Event no. during follow-up	No. at risk[b]	Events	Person-years	Event rate per 100 pyrs (95% CI)		No. at risk[b]	Events	Person-years	Event rate per 100 pyrs (95%CI)
First[c] recurrent stroke	1,034	85	2,222	3.82 (3.09-4.73)		1,034	34	2,330	1.46 (1.04; 2.04)
Second recurrent stroke	85	6	172	3.49 (1.57-7.77)		34	<5	NR[d]	6.61 (2.48-17.60)
**Non-lobar**									
Event no. during follow-up	No. at risk[2]	Events	Person-years	Event rate per 100 pyrs (95% CI)		No. at risk[2]	Events	Person-years	Event rate per 100 pyrs (95% CI)
First[3] recurrent stroke	1,255	37	3,174	1.26 (0.92-1.72)		1,255	52	2,921	1.78 (1.36-2.34)
Second recurrent stroke	37	<5	NR[d]	3.53 (0.88-14.13)		52	NR[d]	85	4.71 (1.77-12.56)

[a]Follow-up was performed separately for intracerebral hemorrhage and ischemic stroke, respectively.
[b]Number of patients available for follow-up of the event being studied, e.g., 122 patients had a first recurrence of ICH and were available for follow-up for a second recurrence of ICH.
[c]Deviates slightly from results of main analysis, as in this analysis follow-up is performed separately for ICH vs IS, whilst in main analysis a common follow-up is used.
[d]Not reported to preserve anonymity in view of small cell counts.

© 2023 Boe NJ initials et al. *JAMA Network Open.*

eTable 9. Baseline characteristics of patients at risk of a second ischemic stroke during follow-up stratified by hematoma location of the index ICH.

	Lobar ICH (n=86)	Non-lobar ICH (n=34)	Crude OR[1] (95% CI)	Age and sex adjusted OR[1] (95% CI)
**Age at baseline (index ICH)**				
Age, mean (SD)	73.5 (10.7)	75.2 (9.8)	Not applicable	Not applicable
**Sex**				
Men	21 (61.8)	29 (55.8)	1 (reference)	1 (reference)
Women	13 (38.2)	23 (44.2)	0.73 (0.36; 1.48)	0.76 (0.37; 1.56)
**Medical history**				
Previous ischemic stroke	6 (17.6)	9 (16.7)	1.19 (0.49; 2.92)	1.16 (0.47; 2.85)
Myocardial infarction	<5[2]	<5[2]	<5[2]	NE[3]
Peripheral artery disease	8 (9.1)	<5[2]	<5[2]	NE[3]
Any of above ('previous occlusive vascular disease')	9 (26.5)	14 (26.9)	1.18 (0.54; 2.55)	1.13 (0.52; 2.47)
Hypertension	27 (79.4)	32 (61.5)	1.40 (0.61; 3.26)	1.38 (0.59; 3.25)
Diabetes	<5	<5	0.60 (0.18; 1.99)	0.58 (0.17; 1.92)
Atrial fibrillation	7 (20.6)	13 (25.0)	0.93 (0.40; 2.16)	0.87 (0.70; 1.07)
COPD	8 (23.5)	14 (26.9)	0.73 (0.33; 1.62)	0.73 (0.33; 1.64)
Diagnoses indicative of high alcohol use	5 (14.7)	7 (13.5)	1.57 (0.60; 4.14)	1.52 (0.56; 4.15)
**Medication before ICH[4]**				
Platelet antiaggregants	14 (41.2)	18 (33.3)	1.76 (0.87; 3.57)	1.66 (0.80; 3.44)
Anticoagulant	<5[2]	10 (18.5)	0.63 (0.22; 1.82)	0.56 (0.19; 1.64)
Statin	14 (41.2)	16 (29.6)	1.73 (0.86; 3.49)	1.55 (0.76; 3.18)
Antihypertensives	15 (44.1)	22 (40.7)	0.97 (0.46; 2.03)	0.89 (0.41; 1.90)

Numbers (percentage), unless otherwise specified.
[1]Odds ratio; comparison of individual covariates as a measure of skewness and using non-lobar ICH as reference group.
[2]Exact numbers not reported to preserve anonymity.
[3]Not estimated due to sparse events.
[4]According to data from Danish National Prescription Registry and corresponding to current use defined as dispensed quantity of drug that lasted until date of onset of index ICH (or ended no later than 30 days before this date).

© 2023 Boe NJ initials et al. *JAMA Network Open.*

eFigure 1. Study flow-chart.

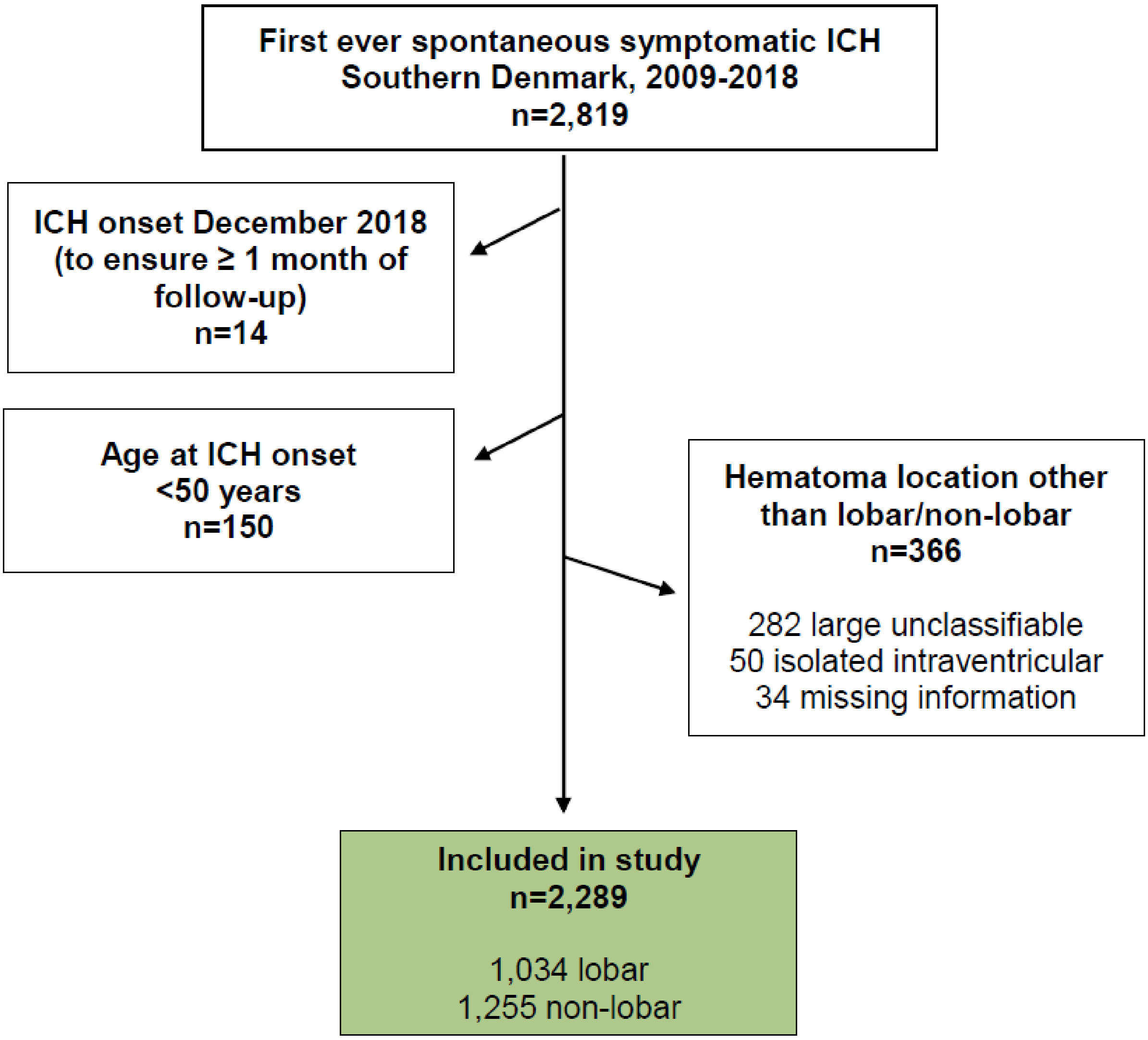

© 2023 Boe NJ initials et al. *JAMA Network Open.*

eFigure 2. Cumulative incidence of main outcomes by ICH location with death as competing risk.

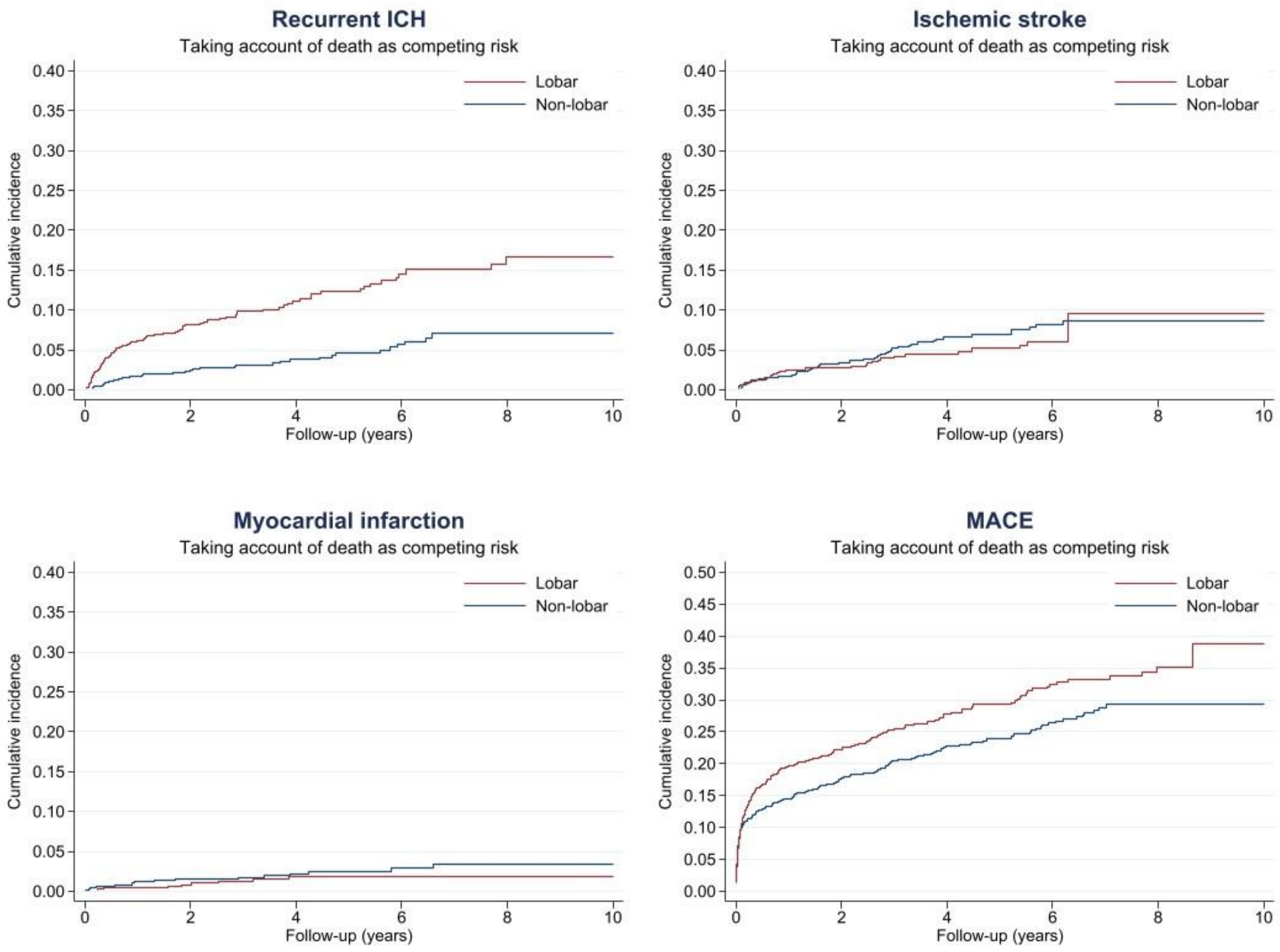

© 2023 Boe NJ initials et al. *JAMA Network Open.*

eFigure 3 Cumulative incidence of main outcomes by ICH location (lobar vs non-lobar) in patients with/without comorbid atrial fibrillation at baseline.

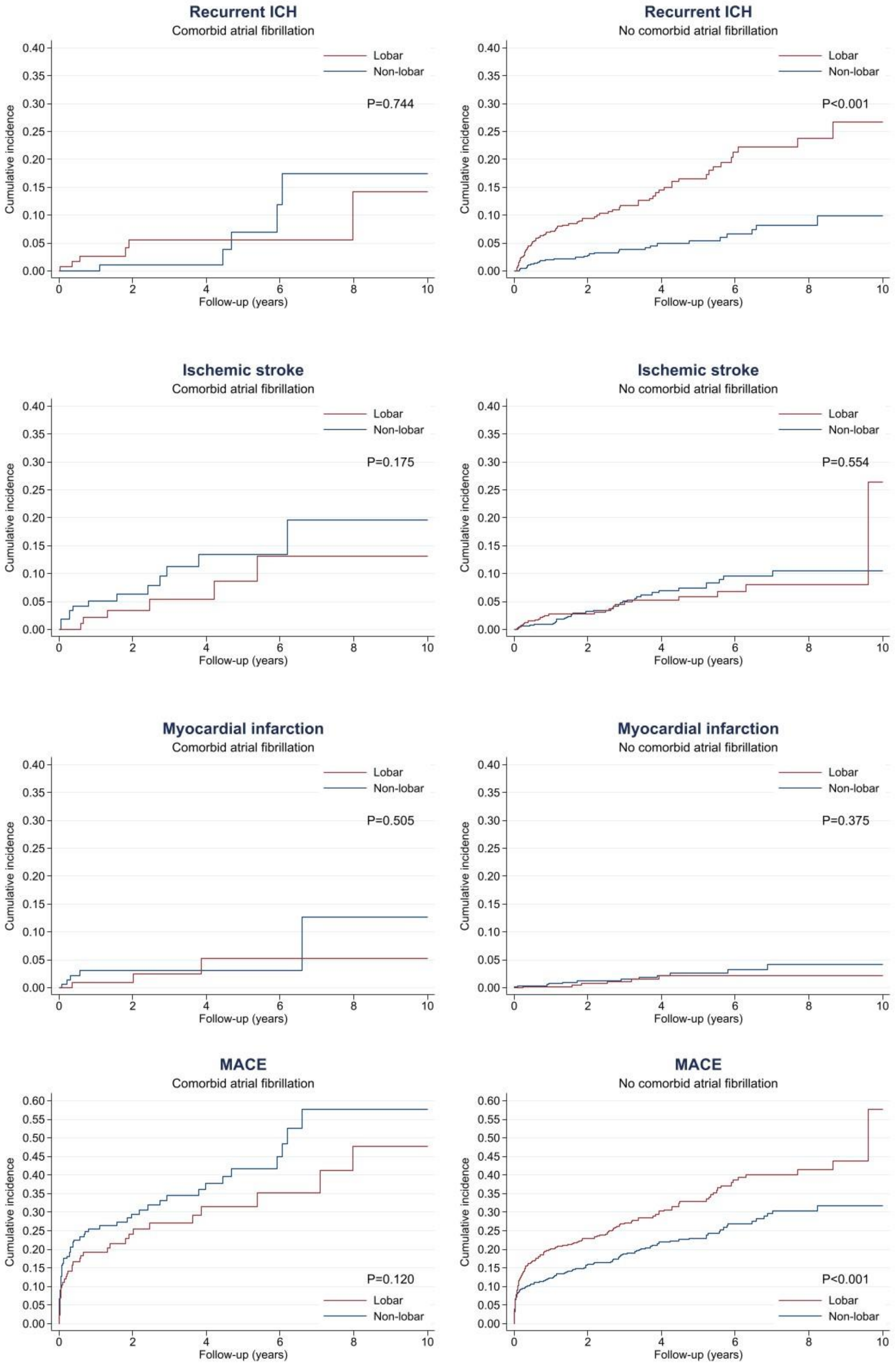

© 2023 Boe NJ initials et al. *JAMA Network Open.*

eFigure 4 Cumulative incidence of main outcomes by ICH location (lobar vs non-lobar) in patients with/without previous occlusive vascular disease at baseline.

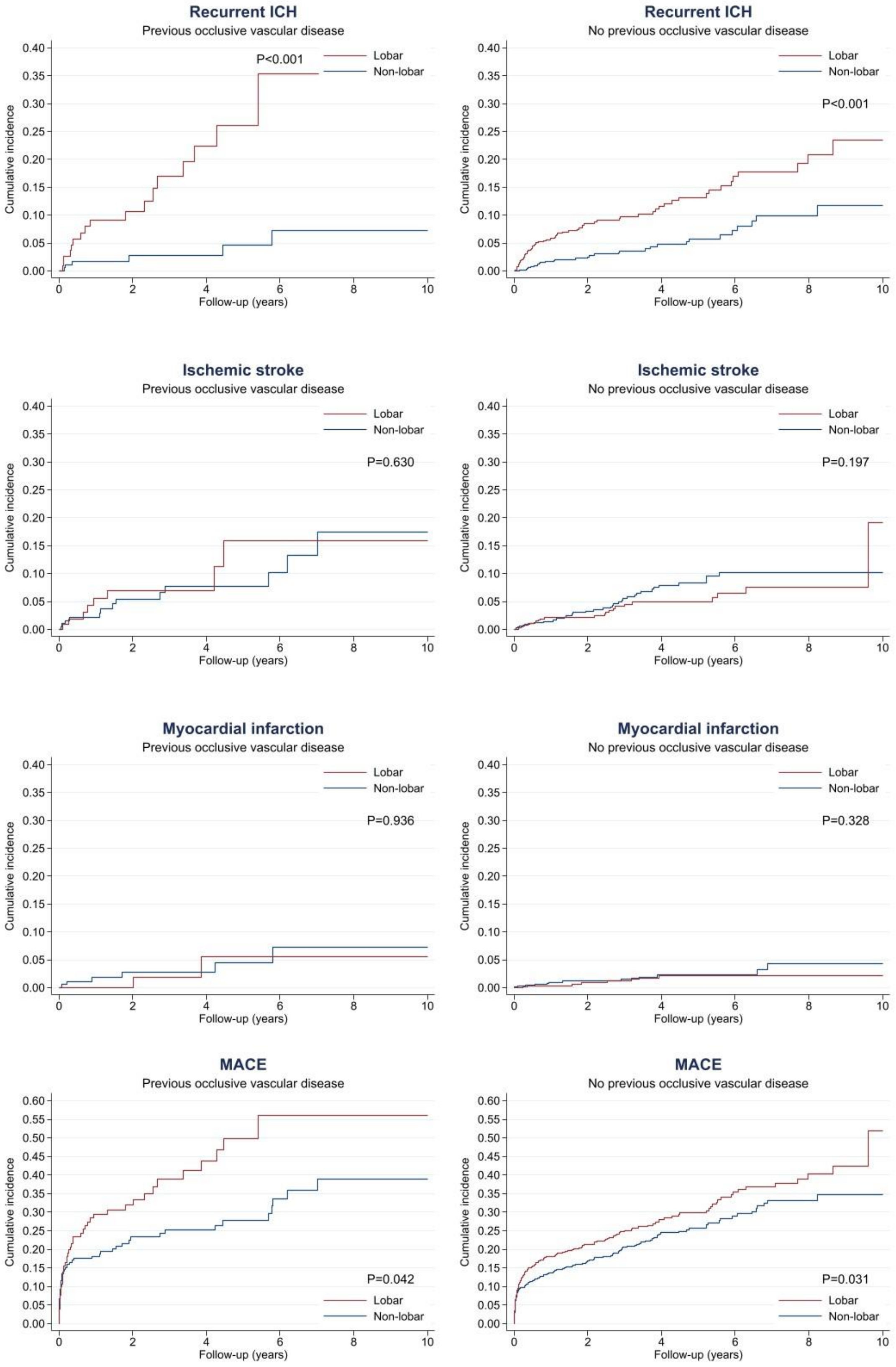

© 2023 Boe NJ initials et al. *JAMA Network Open.*

eFigure 5 Annual incidence rate (95% confidence intervals indicated with bars) of main outcomes for lobar ICH and non-lobar ICH.

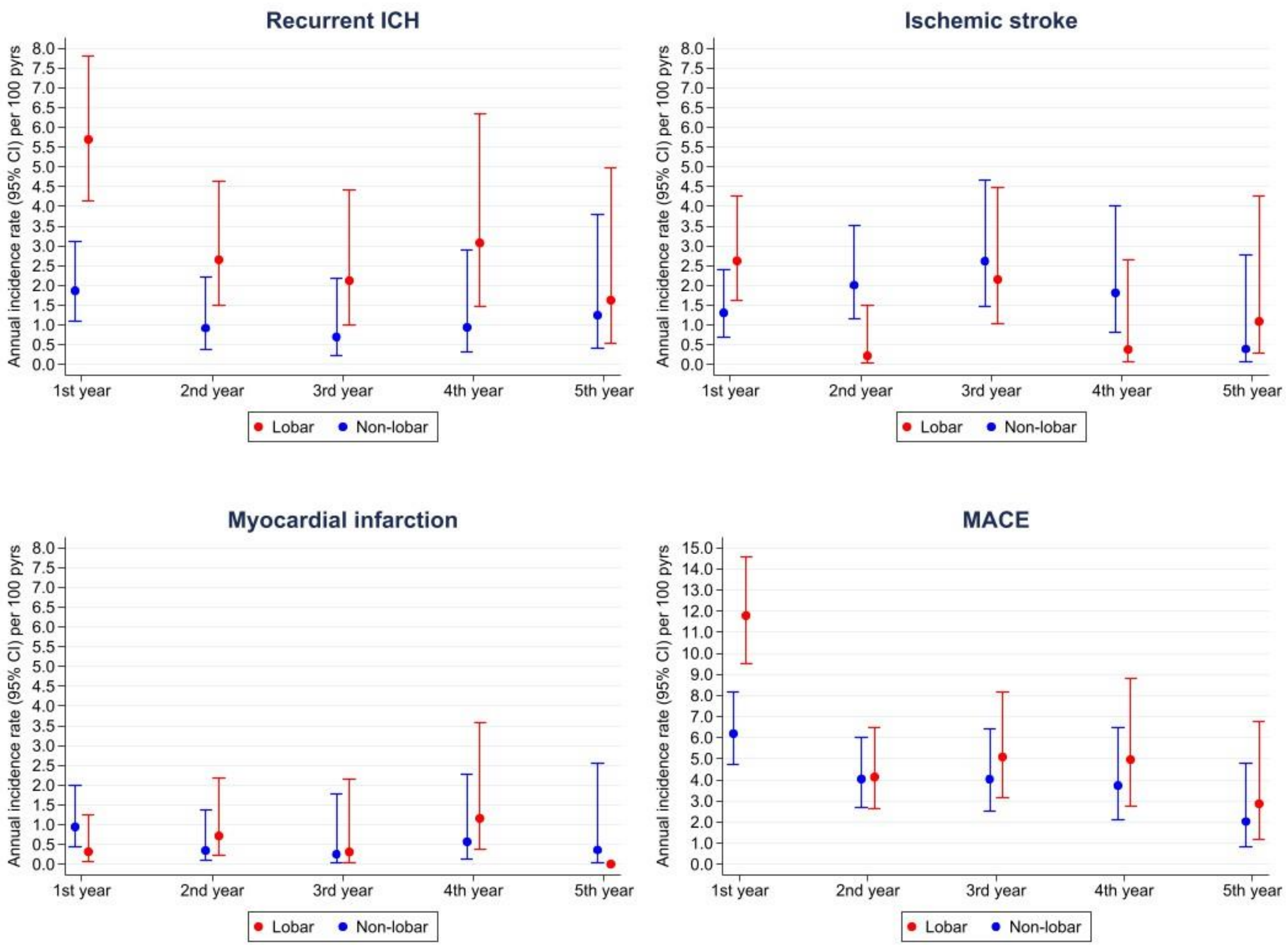

© 2023 Boe NJ initials et al. *JAMA Network Open.*
