# Supplementary material for: Major Cardiovascular Events After Spontaneous Intracerebral Hemorrhage by Hematoma Location
Source: JAMA Netw Open. 2023 Apr 5;6(4):e235882. doi: 10.1001/jamanetworkopen.2023.5882 (PMC10077102; doi:10.1001/jamanetworkopen.2023.5882)
Supplement: Supplement 2. — Data Sharing Statement [file jamanetwopen-e235882-s002.pdf]

Data Sharing Statement

Boe. Major Cardiovascular Events After Spontaneous Intracerebral Hemorrhage by Hematoma Location. *JAMA Netw Open*. Published April 05, 2023.
doi:10.1001/jamanetworkopen.2023.5882

Data
Data available: No

Additional Information
Explanation for why data not available: Danish law prohibits the authors from sharing or granting access to the data used for this study.
